# Hepatitis B virus X protein induces ALDH2 ubiquitin-dependent degradation to enhance alcoholic steatohepatitis

**DOI:** 10.1093/gastro/goad006

**Published:** 2023-03-01

**Authors:** Haoxiong Zhou, Sizhe Wan, Yujun Luo, Huiling Liu, Jie Jiang, Yunwei Guo, Jia Xiao, Bin Wu

**Affiliations:** Department of Gastroenterology, The Third Affiliated Hospital of Sun Yat-sen University, Guangzhou, Guangdong, P. R. China; Alcoholic Liver Disease Center, The Third Affiliated Hospital of Sun Yat-sen University, Guangzhou, Guangdong, P. R. China; Guangdong Provincial Key Laboratory of Liver Disease Research, Guangzhou, Guangdong, P. R. China; Department of Gastroenterology, The Third Affiliated Hospital of Sun Yat-sen University, Guangzhou, Guangdong, P. R. China; Alcoholic Liver Disease Center, The Third Affiliated Hospital of Sun Yat-sen University, Guangzhou, Guangdong, P. R. China; Guangdong Provincial Key Laboratory of Liver Disease Research, Guangzhou, Guangdong, P. R. China; Department of Gastroenterology, The Third Affiliated Hospital of Sun Yat-sen University, Guangzhou, Guangdong, P. R. China; Alcoholic Liver Disease Center, The Third Affiliated Hospital of Sun Yat-sen University, Guangzhou, Guangdong, P. R. China; Guangdong Provincial Key Laboratory of Liver Disease Research, Guangzhou, Guangdong, P. R. China; Department of Gastroenterology, The Third Affiliated Hospital of Sun Yat-sen University, Guangzhou, Guangdong, P. R. China; Alcoholic Liver Disease Center, The Third Affiliated Hospital of Sun Yat-sen University, Guangzhou, Guangdong, P. R. China; Guangdong Provincial Key Laboratory of Liver Disease Research, Guangzhou, Guangdong, P. R. China; Department of Gastroenterology, The Third Affiliated Hospital of Sun Yat-sen University, Guangzhou, Guangdong, P. R. China; Alcoholic Liver Disease Center, The Third Affiliated Hospital of Sun Yat-sen University, Guangzhou, Guangdong, P. R. China; Guangdong Provincial Key Laboratory of Liver Disease Research, Guangzhou, Guangdong, P. R. China; Department of Gastroenterology, The Third Affiliated Hospital of Sun Yat-sen University, Guangzhou, Guangdong, P. R. China; Alcoholic Liver Disease Center, The Third Affiliated Hospital of Sun Yat-sen University, Guangzhou, Guangdong, P. R. China; Guangdong Provincial Key Laboratory of Liver Disease Research, Guangzhou, Guangdong, P. R. China; Clinial Medical Research Institute, The First Affiliated Hospital of Jinan University, Guangzhou, Guangdong, P. R. China; Department of Metabolic and Bariatric Surgery, The First Affiliated Hospital of Jinan University, Guangzhou, Guangdong, P. R. China; Department of Gastroenterology, The Third Affiliated Hospital of Sun Yat-sen University, Guangzhou, Guangdong, P. R. China; Alcoholic Liver Disease Center, The Third Affiliated Hospital of Sun Yat-sen University, Guangzhou, Guangdong, P. R. China; Guangdong Provincial Key Laboratory of Liver Disease Research, Guangzhou, Guangdong, P. R. China

**Keywords:** hepatitis B virus X protein, alcoholic steatohepatitis, acetaldehyde dehydrogenase 2, ubiquitination, lysophospholipids, reactive oxygen species

## Abstract

**Background:**

Excessive alcohol intake with hepatitis B virus (HBV) infection accelerates chronic liver disease progression and patients with HBV infection are more susceptible to alcohol-induced liver disease. Hepatitis B virus X protein (HBx) plays a crucial role in disease pathogenesis, while its specific role in alcoholic liver disease (ALD) progression has not yet been elucidated. Here, we studied the role of HBx on the development of ALD.

**Methods:**

HBx-transgenic (HBx-Tg) mice and their wild-type littermates were exposed to chronic plus binge alcohol feeding. Primary hepatocytes, cell lines, and human samples were used to investigate the interaction between HBx and acetaldehyde dehydrogenase 2 (ALDH2). Lipid profiles in mouse livers and cells were assessed by using liquid chromatography–mass spectrometry.

**Results:**

We identified that HBx significantly aggravated alcohol-induced steatohepatitis, oxidative stress, and lipid peroxidation in mice. In addition, HBx induced worse lipid profiles with high lysophospholipids generation in alcoholic steatohepatitis, as shown by using lipidomic analysis. Importantly, serum and liver acetaldehyde were markedly higher in alcohol-fed HBx-Tg mice. Acetaldehyde induced lysophospholipids generation through oxidative stress in hepatocytes. Mechanistically, HBx directly bound to mitochondrial ALDH2 to induce its ubiquitin–proteasome degradation, resulting in acetaldehyde accumulation. More importantly, we also identified that patients with HBV infection reduced ALDH2 protein levels in the liver.

**Conclusions:**

Our study demonstrated that HBx-induced ubiquitin-dependent degradation of mitochondrial ALDH2 aggravates alcoholic steatohepatitis.

## Graphical Abstract

**Graphical Abstract:**
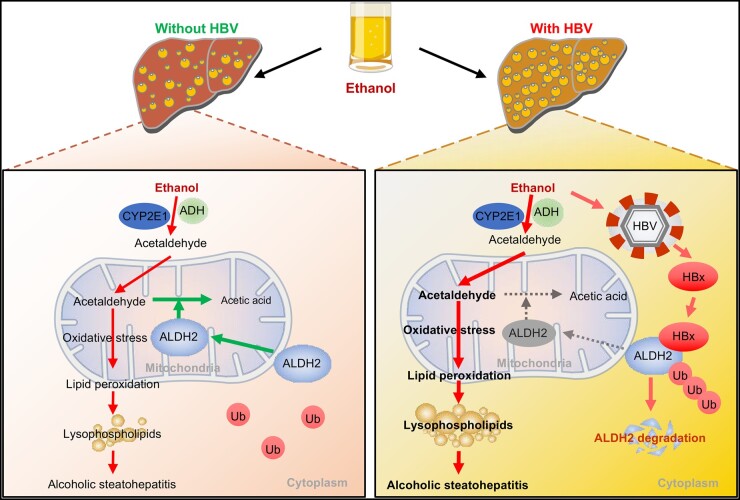

## Introduction

Alcoholic liver disease (ALD), which is caused by chronic and excessive alcohol intake, is a major cause of chronic liver disease worldwide [1, 2]. A wide variety of environmental and host factors and co-morbidities have been shown to influence the development of ALD, including gender, drinking pattern, genetic factors, age, and chronic viral hepatitis [3]. Among these risk factors, hepatitis B virus (HBV) infection is the major cause of chronic liver disease worldwide, with ∼257 million individuals chronically infected [4]. A great number of individuals suffer from concurrent ALD and HBV infection [5]. In our previous study [6], we found that ALD patients with combined HBV infection developed more severe liver impairment and cirrhosis complications than ALD patients without HBV infection. However, why patients with HBV infection are more susceptible to alcohol-induced liver disease is still largely obscure.

Hepatitis B virus X protein (HBx), encoded by the HBV genome, is a multifunctional viral regulator involved in viral pathogenesis, such as DNA repair inhibition [7], cell cycle arrest [8], and posttranscriptional regulation [9, 10]. HBx induces lipid accumulation by activating sterol regulatory element-binding protein 1 (SREBP1) and peroxisome proliferator-activated receptor gamma (PPAR-γ) in liver cells, leading to HBV-associated hepatic steatosis [11]. A recent study reported that chronic ethanol consumption and HBV cooperatively induced disrupted lipid metabolism by HBx/SWELL1/arachidonic acid signaling in the liver [12]. These studies highlight the importance of HBx in HBV-mediated abnormal lipid metabolism and suggest that HBx may play a crucial role in disease progression in alcoholics with HBV infection. In addition, HBx was reported to sensitize primary mouse hepatocytes to ethanol-induced apoptosis [13]. However, the *in vivo* role of HBx in alcohol-induced liver injury is still unclear.

Acetaldehyde has been increasingly identified as a primary culprit in the development of ALD given its role in forming a variety of proteins and DNA adducts to impair cellular function, deplete mitochondrial glutathione, and promote oxidative stress and lipid peroxidation [14, 15]. Acetaldehyde dehydrogenase 2 (ALDH2) is the most potent enzyme responsible for acetaldehyde clearance [16]. Considering the important function of mitochondrial ALDH2 in ALD progression, the mechanisms underlying the regulation of ALDH2 activity and stability are worthy of investigation.

Perturbed lipid homeostasis results in steatosis, lipid peroxidation, inflammation, and fibrotic processes that are important pathophysiological determinants in ALD pathogenesis [17]. Advances in lipidomic analysis now permit simultaneous quantitative measurement of individual lipid species among all major lipid classes from biological samples. The levels of lysophospholipids (lysoPLs), a class of bioactive lipid mediators, have been reported to be elevated in diverse diseases, including ALD [18–20]. Here, we found that HBx aggravated alcoholic steatohepatitis, by using a model of HBx-transgenic (Tg) mice. Mechanistically, HBx directly interacted with mitochondrial ALDH2 and promoted ALDH2 ubiquitination and degradation, thereby inducing acetaldehyde accumulation. Notably, excessive acetaldehyde induced lipid peroxidation and lysophospholipids production. We also confirmed that the expression of ALDH2 was reduced in HBV-infected human liver. These results have broad implications for management of ALD patients with concurrent HBV infection and provide a convincing rationale for alcohol abstinence in HBV-infected patients.

## Materials and methods

### Human samples

Human liver samples were collected from individuals who underwent liver biopsy, liver surgery, or liver transplantation in the clinic at the Third Affiliated Hospital of Sun Yat-sen University (Guangzhou, China). These samples were divided into two groups: the non-HBV-infected group (*n *=* *10) and the HBV-infected group (*n *=* *10). In the HBV-infected group, all patients were HBsAg-positive with detectable HBV DNA and none of the patients included in the study was taking antiviral therapy. Patients with inactive ALDH2*2 mutation, cirrhosis, and other viral infections except HBV were excluded from this study. The patient characteristics are shown in Supplementary Table S1. All procedures that involved human sample collection were approved by the ethics committee of the Third Affiliated Hospital of Sun Yat-sen University (No. [2019] 02–248-01). Informed consent was obtained from all included patients.

### Animal model

All animals were housed with water and food available ad libitum in a specific pathogen-free room on a 12-h light/dark cycle with temperature control (21 ± 1°C). HBx-Tg mice (C57BL/6 background) and wild-type (WT) littermates were generated as previously described [21]. Female mice at 12 weeks old were used in this study. For *in vivo* ethanol feeding studies, WT and HBx-Tg mice were subjected to chronic-binge ethanol feeding (Gao-Binge model) [22]. In brief, mice were fed the Lieber–DeCarli diet (Dyets, Bethlehem, USA) containing 5% (vol/vol) ethanol for 10 d and gave a single dose of ethanol (5 g/kg body weight) in the early morning on Day 11. Pair-fed control mice received a diet with an isocaloric substitution of dextrose. All mice were sacrificed 9 h after the final binge. Livers were weighed and serum samples were obtained for further experiments. All animal experiments followed protocols approved by the Institutional Animal Care and Use Committee at the Third Affiliated Hospital of Sun Yat-sen University (No. 00283501).

### Cell culture and treatments

The human hepatoma cell lines HepG2.215 and HepG2-NTCP and the human normal hepatocyte cell line LO2 were used in this study, and were maintained in Dulbecco’s modified Eagle’s medium (DMEM) (Thermo Fisher Scientific, MA, USA) supplemented with 10% fetal bovine serum (FBS) (CellCook, Guangzhou, China), 100 U/mL penicillin, and 100 mg/mL streptomycin. HepG2.215 and LO2 cell lines were provided by the Guangdong Provincial Key Laboratory of Liver Disease Research, China, as previously described [21]. The HepG2-NTCP cell line was kindly provided by Professor Liang Peng at the Third Affiliated Hospital of Sun Yat-sen University. All cells were cultured at 37°C in a humidified 5% CO_2_ incubator.

For ethanol and acetaldehyde treatment, hepatocytes were treated with fresh culture medium containing the indicated concentrations of ethanol and acetaldehyde for 48 h. Considering the high evaporation ability of ethanol and acetaldehyde, the medium was refreshed every 6 h. To inhibit acetaldehyde-induced reactive oxygen species (ROS), cells were treated with 10 mM N-Acetylcysteine (NAC) (MedChemExpress, NJ, USA) for 48 h. For Cycloheximide (CHX) chasing assays, cells were treated with 100 μg/mL CHX (MedChemExpress, NJ, USA) for the indicated times. To inhibit proteasomal degradation, cells were treated with 20 μM MG132 (MedChemExpress, NJ, USA) for 6 h.

HBV infection of HepG2-NTCP cells was performed as previously reported [10]. The HBV-containing culture medium of HepG2.215 cells were collected and concentrated polyethylene glycol precipitation (PEG) was performed. HepG2-NTCP cells were seeded in collagen-coated plates and infected with HBV (MOI 500 genome equivalents/cell) with 4% PEG 8000 and 2% dimethyl sulfoxide (DMSO) for 24 h. After incubation with HBV particles, the cells were washed three times with phosphate buffered saline (PBS) and maintained in DMEM with 10% FBS and 2% DMSO. Infected cells were incubated for at least 5 d and the medium was changed every 2 d.

### Isolation and culture of primary mouse hepatocytes

Primary mouse hepatocytes were isolated from 12-week-old WT and HBx-Tg female mice using two-step collagenase perfusion and low-speed gradient centrifugation methods as previously described [23]. Briefly, mice were anesthetized and perfused with buffer solution lacking Ca^2+^ for 10 min and then perfused with 0.05% type IV collagenase (Sigma–Aldrich, St. Louis, MO, USA) through the portal vein. After perfusion, the liver was minced and filtered through a 70-μm filter. Then hepatocytes were dissociated by two cycles of centrifugation at 50 *g* for 2 min. The obtained hepatocytes were resuspended in DMEM with 10% FBS and 1% ITS and seeded in plates coated with collagen (Sigma–Aldrich, St. Louis, MO, USA) prior to further experiments.

### Histological analysis

Liver samples were fixed in 4% paraformaldehyde and routinely dehydrated, transparentized, waxed, and paraffin-embedded. Hematoxylin and eosin (H&E) staining was performed on paraffin-embedded sections to visualize the pattern of inflammation status and lipid accumulation. Oil Red O staining of frozen liver sections was performed to visualize intracellular lipid droplets. The non-alcoholic fatty liver disease (NAFLD) activity score (NAS) of each group was calculated as previously described [24]. Representative images from both groups are shown. Image processing and quantification analysis were performed using ImageJ (https://imagej.nih.gov/ij/).

### Serological analysis and enzyme-linked immunosorbent assay

Serum alanine aminotransferase (ALT), aspartate aminotransferase (AST), triglyceride (TG), and total cholesterol (TC) of mice were then measured using an Olympus AU5400 clinical biochemical analyser (Tokyo, Japan). Concentrations of mouse IL-1β, TNF-α, and VCAM1 were detected using corresponding serological analysis and enzyme-linked immunosorbent assay (ELISA) kits (Elabscince, Wuhan, China) according to the manufacturer’s instructions.

### Immunohistochemistry and immunofluorescence

For immunohistochemistry (IHC), paraffin-embedded sections were routinely dewaxed and hydrated. Subsequently, 0.3% hydrogen peroxide was utilized to inactivate endogenous peroxidase. After antigen retrieval, the sections were blocked with 5% goat serum albumin for 1 h at 37°C. Then the sections were incubated with rabbit anti-4-HNE (Abcam, Cambridge, MA, USA) at 4°C overnight, followed by a 60-min incubation with goat anti-rabbit IgG (Abcam, Cambridge, MA, USA). The positive signals were detected by using a diaminobenzidine (DAB) detection kit (Vector, Burlingame, CA, USA) according to the manufacturer’s instructions, followed by counterstaining with hematoxylin and cover slipping with Permount (Thermo Fisher Scientific, MA, USA). To detect liver ROS production, frozen liver sections were stained with dihydroethidium (DHE) (Beyotime, Shanghai, China) for 30 min at 37°C. The nuclei were stained with 4',6-diamidino-2-phenylindole (DAPI) (Abcam, Cambridge, MA, USA). Images were obtained using a fluorescence microscope (Carl Zeiss, Germany). Raw TIF files were processed using ImageJ to create stacks, adjust levels, and apply false coloring.

### Cell transfection

The full-length (1–154 aa) and truncated HBx (51–154, 1–50/101/154, and 1–100 aa) were cloned into pcDNA3.1(+)-HA vectors. Full-length human ALDH2 was cloned into the pcDNA3.1(+)-FLAG vector. LO2 cells were cultured in a 6-well or 24-well plate for 24 h and then transfected with plasmids or siRNAs. The transfections were performed using Lipofectamine 3000 reagent (Invitrogen, MA, USA) according to the manufacturer’s instructions. Further experiments were performed 48 h after transfection. The siRNA duplex sequences used were as follows:

si-HBx, GCGGGACGUCCUUUGUUUA;

si-NC, UUCUCCGAACGUGUCACGU.

### Mitochondrial fractionation

Mitochondrial fractionation was performed using the Mitochondrial Isolation Kit for Cultured Cells (Thermo Fisher Scientific, MA, USA) following the manufacturer’s instructions. In brief, 1 × 10^7^ cells were collected and incubated in mitochondria isolation buffers on ice for 5 min. After homogenization, nuclei and intact cells were discarded after centrifugation at 700 *g* for 10 min at 4°C. The supernatant enriched with the cytosolic fraction and the pellet enriched with the mitochondrial fraction were obtained after centrifugation at 12,000 *g* for 15 min at 4°C. For mitochondrial protein-localization assays as previously described [25], the purified mitochondria were divided into three groups. The first group was not treated, as a control. The second group was treated with 3 μM proteinase K (Invitrogen, MA, USA). The third group was treated with 3 μM proteinase K and 0.1% Triton X-100 solution. Three groups of samples were placed in a 37°C water bath for 30 min. All samples were prepared for further immunoblotting.

### Liquid chromatography–mass spectrometry/mass spectrometry lipidomic analysis

Lipid extracts from samples were prepared using a methyl-tert-butyl ether (MTBE) extraction procedure [26]. In brief, ∼20 mg of liver tissue was homogenized with 1 mL of mixture (including methanol, MTBE, and internal standard mixture) and steel balls. The steel balls were removed and the mixture was swirled for 15 min. Then, 200 μL of water was added and the mixture was swirled for 1 min and centrifuged at 12,000 r/min at 4°C for 10 min. After centrifugation, the supernatant containing the total lipid extract (TLE) was transferred to a vial and dried under N_2_ gas. The TLE was then redissolved in acetonitrile/isopropanol (10:90, vol/vol) with 0.1% formic acid and 10 mmol/L ammonium formate before being subjected to liquid chromatography–mass spectrometry (LC–MS)/MS analysis as described below. For cell samples, ∼1 × 10^7^ cells were collected and flash-frozen in liquid nitrogen. The other steps were the same as those used for the tissue extraction method.

Multiple reaction monitoring (MRM) profiling of the extracted lipids was performed on an LC–electrospray ionization (ESI)–MS/MS system (SCIEX, CA). The analytical conditions have been described elsewhere [20]. The effluent was alternatively connected to an ESI-triple quadrupole-linear ion trap (QTRAP)–MS. Triple quadrupole (QQQ) scans were performed on a triple quadrupole-linear ion trap mass spectrometer (QTRAP) with a QTRAP^®^ 6500+ LC–MS/MS System, equipped with an ESI Turbo Ion-Spray interface, operating in positive and negative ion mode and controlled by Analyst 1.6.3 software (Sciex). Instrument tuning and mass calibration were performed with 10-μmol/L polypropylene glycol solutions in QQQ mode. QQQ scans were acquired as MRM experiments with collision gas (nitrogen) set to 34.5 KPa. A specific set of MRM transitions was monitored for each period according to the metabolites eluted within this period. Lipid contents were detected based on the AB Sciex QTRAP 6500 LC–MS/MS platform.

### Western blot and co-immunoprecipitation

Tissues and cells were lysed with radio-immunoprecipitation assay buffer (Thermo Fisher Scientific, MA, USA) with protease inhibitor cocktail. The protein concentration was measured using a BCA Protein Assay Kit (Thermo Fisher Scientific, MA, USA). Equal amounts of protein samples were separated by 10% sodium dodecyl sulfate (SDS)–polyacrylamide gel electrophoresis and transferred to nitrocellulose membranes. After blocking with 5% non-fat milk, blots were probed overnight at 4°C with 1:4,000 GAPDH (Cell Signaling Technology, MA, USA), 1:100 HBx (Santa Cruz, CA, USA), 1:1,000 alcohol dehydrogenase (ADH) (Abcam, Cambridge, MA, USA), 1:2,000 CYP2E1 (Proteintech, Cat#19937–1-AP), 1:1,000 ALDH1A1 (Proteintech, Rosemont, USA), 1:1,000 ALDH1B1 (Proteintech, Rosemont, USA), 1:1,000 ALDH2 (Proteintech, Rosemont, USA), 1:4,000 COXIV (Cell Signaling Technology, MA, USA), 1:1,000 Tom20 (Proteintech, Rosemont, USA), 1:1,000 Tim23 (Proteintech, Rosemont, USA), 1:200 Ubiquitin (Santa Cruz, CA, USA), 1:2,000 HA (Cell Signaling Technology, MA, USA), and 1:2,000 Flag (Sigma–Aldrich, St. Louis, MO, USA). Then, the blots were incubated with horse radish peroxidase (HRP)-conjugated secondary antibodies for 2 h at room temperature and developed using enhanced chemiluminescence (ECL) detection reagents.

For co-immunoprecipitation, cells were lysed in immunoprecipitation (IP) lysis buffer (Thermo Fisher Scientific, MA, USA) with a protease inhibitor cocktail on ice for 30 min. Cell lysates were centrifuged at 12,000 *g* for 10 min and the supernatants were incubated with the indicated antibodies absorbed to protein A/G magnetic beads (MedChemExpress, NJ, USA) overnight at 4°C and eluted from beads with SDS loading buffer at 95°C for 10 min after washing three times with PBST (0.1% Tween-20). The eluted samples were then subjected to immunoblot analysis. IgG antibodies (Beyotime, Shanghai, China) from the corresponding species were used as a control.

### *In vivo* ubiquitination analysis

For ubiquitination analysis, cells were lysed with IP lysis buffers containing 1% SDS mixed with protease inhibitor cocktail. After denaturation at 95°C for 10 min, the lysates were diluted 10-fold with IP lysis buffers and incubated for 30 min at 4°C, and centrifuged at 12,000 *g* for 20 min at 4°C. The next steps were the same as those used for co-IP.

### RNA extraction and real-time polymerase chain reaction (PCR)

Total RNA was extracted from cells and tissues using TRIzol reagent (Invitrogen, MA, USA). cDNA was reverse transcribed with the PrimeScript™ II 1st Strand cDNA Synthesis Kit (TaKaRa, Japan). Real-time PCR was performed with a real-time PCR System (BioRad, CA) using gene-specific primers and SYBR Green (Vazyme, Nanjing, China). The GAPDH mRNA levels were used for normalization. Experiments were conducted in three independent duplicates. All primers used are listed in Supplementary Table S2.

### Detection of HBV gene expression and replication

The level of HBV DNA in the culture medium was quantified by using the COBAS^®^ TaqMan 48^®^ assay (Roche, Switzerland) as previously described [21]. The HBsAg and HBeAg levels in the culture supernatants were measured by using an HBsAg ELISA Kit (Kehua Bio-engineering, Shanghai, China) and HBeAg ELISA Kit (Kehua Bio-engineering, Shanghai, China), respectively, according to the manufacturer’s protocols. Total RNA was extracted from cells with TRIzol reagent. HBV pgRNA and HBx mRNA were measured by using real-time PCR using the specific primers listed in Supplementary Table S2.

### Measurement of ethanol and acetaldehyde

Serum and liver ethanol and acetaldehyde levels were determined by using gas chromatograph–mass spectrometry (GC–MS) (Agilent Technologies, Santa Clara). After adding the corresponding internal standard, the serum and liver homogenates were centrifuged at 12,000 *g* for 15 min at 4°C. Then, the supernatant was quantitatively transferred into a sealed vial. The serum and liver samples were then subjected to GC–MS analysis.

### Measurement of ADH and ALDH activity

ADH and ALDH activities were measured by using an alcohol dehydrogenase activity assay kit (Boxbio, Beijing, China) and acetaldehyde dehydrogenase activity assay kit (Boxbio, Beijing, China), respectively, following the manufacturer’s instructions.

### Total ROS, glutathione, oxidative glutathione, superoxide dismutase activity, and malonaldehyde measurements

For cellular ROS detection, cells were incubated with 10 μM of 2,7-Dichlorodihydrofluorescein diacetate (DCFH-DA) (MedChemExpress, NJ, USA) working solution at 37°C for 20 min in darkness. The fluorescence light intensity was measured by using a multifunction microplate reader (Infinite 200 Pro, Tecan, Australia). Cellular glutathione (GSH), oxidative glutathione (GSSG), superoxide dismutase (SOD) activity, and malonaldehyde (MDA) levels were measured by using the corresponding commercial assay kits (Boxbio, Beijing, China), according to the manufacturer’s instructions.

### Molecular docking simulation

The structure of ALDH2 was obtained from a Protein Data Bank file (https://www.rcsb.org/; accession number: 1cw3). The structure of HBx was predicted by applying the state-of-the-art machine-learning method, AlphaFold2 [27]. Structural analysis and attribution of the residue interaction networks between HBx and ALDH2 were analysed and visualized by using PYMOL software (http://pymol.org/2/).

### Statistical analysis

All statistical analyses were performed using GraphPad Prism (version 8.0). Replicates are described in the figure legends. The data are presented as the mean ± standard deviation (SD). Data were analysed by two-tailed Student’s *t*-test for comparisons of two groups, one-way ANOVA with Tukey’s post-test for univariate comparisons of multiple groups and two-way ANOVA with Bonferroni’s multiple comparison post-test for bivariate comparisons of multiple groups. The statistical parameters are shown in the figure legends where *P*-values of <0.05 were considered to represent a statistically significant difference. All experiments were repeated independently at least three times.

## Results

### HBx aggravates ethanol-induced liver injury and steatohepatitis in mice

**Figure 1.**
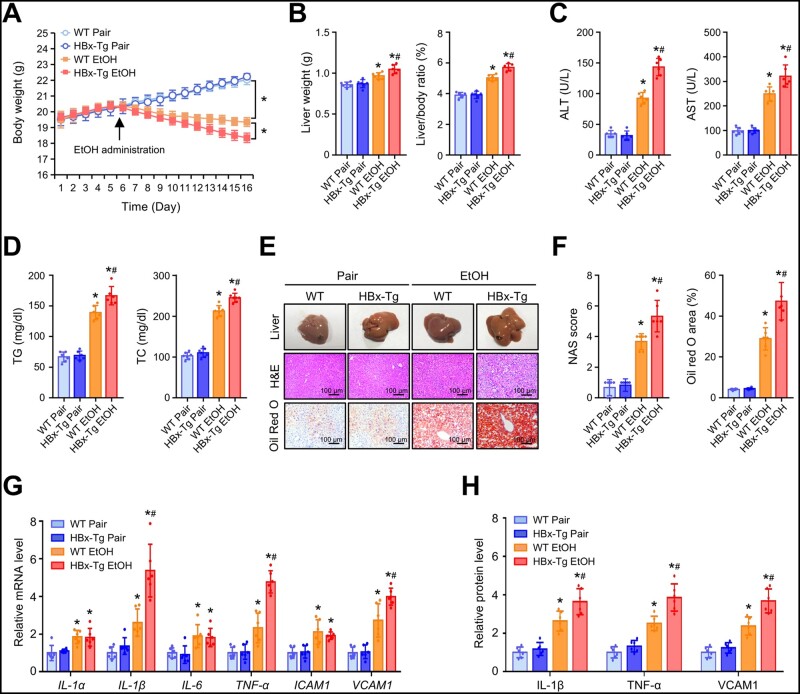
HBx aggravates ethanol-induced liver injury and steatohepatitis in mice. (A) Body weight changes of WT and HBx-Tg mice fed a control diet or ethanol diet. (B) Liver weight and ratios of liver weight to body weight of mice in the indicated groups. (C) Serum ALT and AST levels of mice in the indicated groups. (D) Serum TG and TC levels of mice in the indicated groups. (E) Representative macroscopic liver photographs (upper), hematoxylin and eosin (H&E) (middle), and Oil Red O (lower) staining of the liver sections. (F) NAS score and percentage of Oil Red O-positive area of the liver sections. (G) Relative mRNA levels of proinflammatory cytokines in the livers of mice in the indicated groups. (H) Relative protein levels of IL-1β, TNF-α, and VCAM1 in the serum of mice in the indicated groups. Data are shown as the mean ± SD (*n *=* *6 in each group). For (A), ^*^P <* *0.05 by two-way ANOVA with Bonferroni’s multiple comparison post-test. For (B)–(D) and (F)–(H), ^*^P <* *0.05 compared with the respective pair-fed group; ^#^*P *<* *0.05 compared with the WT ethanol-fed group by one-way ANOVA with Tukey’s multiple comparison post-test. EtOH, ethanol; ALT, alanine aminotransferase; AST, aspartate aminotransferase; TG, triglyceride; TC, total cholesterol.

First, we examined the effect of ethanol (EtOH) on HBV replication in HepG2.215 cells with HBV DNA integration [28]. As expected, we found that EtOH promoted HBV replication and HBV gene expression as well as HBx overexpression in a dose-dependent manner in HepG2.215 cells (Supplementary Figure S1). To investigate the effect of HBx on alcoholic steatohepatitis, we fed WT mice and HBx-Tg mice a Lieber–DeCarli liquid diet containing 5% (vol/vol) ethanol for 10 d and administered a single dose of ethanol (5 g/kg) on Day 11. During an 11-day ethanol consumption period, HBx-Tg mice showed a marked reduction in body weight compared with the WT mice (Figure 1A). Diet consumption was comparable in both dietary groups. In contrast, the liver weight and liver-to-body ratio were higher in ethanol-fed HBx-Tg mice than in ethanol-fed WT mice (Figure 1B). Chronic-binge ethanol consumption significantly increased serum alanine aminotransferase (ALT), serum aspartate aminotransferase (AST), serum triglyceride (TG), and serum TC in WT mice, but the effect was much greater in HBx-Tg mice (Figure 1C and D). Histologically, ethanol-fed HBx-Tg mice, in comparison to WT mice fed the same diet, exhibited more lipid accumulation and inflammatory cell infiltration in the liver, as indicated by H&E and Oil Red O staining (Figure 1E and F). Moreover, chronic-binge ethanol consumption led to significantly increased expression of genes related to pro-inflammatory cytokines, including *IL-1α*, *IL-1β*, *IL-6*, *TNF-α*, *ICAM-1*, and *VCAM-1*. Among these genes, ethanol-fed HBx-Tg mice had higher levels of *IL-1β*, *TNF-1*, and *VCAM-1* in the liver (Figure 1G). Accordingly, the serum concentrations of IL-1β, TNF-α, and VCAM-1 in HBx-Tg mice were also significantly higher than those in WT mice (Figure 1H). These results indicate that HBx-Tg mice are more susceptible to ethanol-induced liver injury and steatohepatitis.

### HBx upregulates ethanol-induced lysophospholipids generation in mice

**Figure 2.**
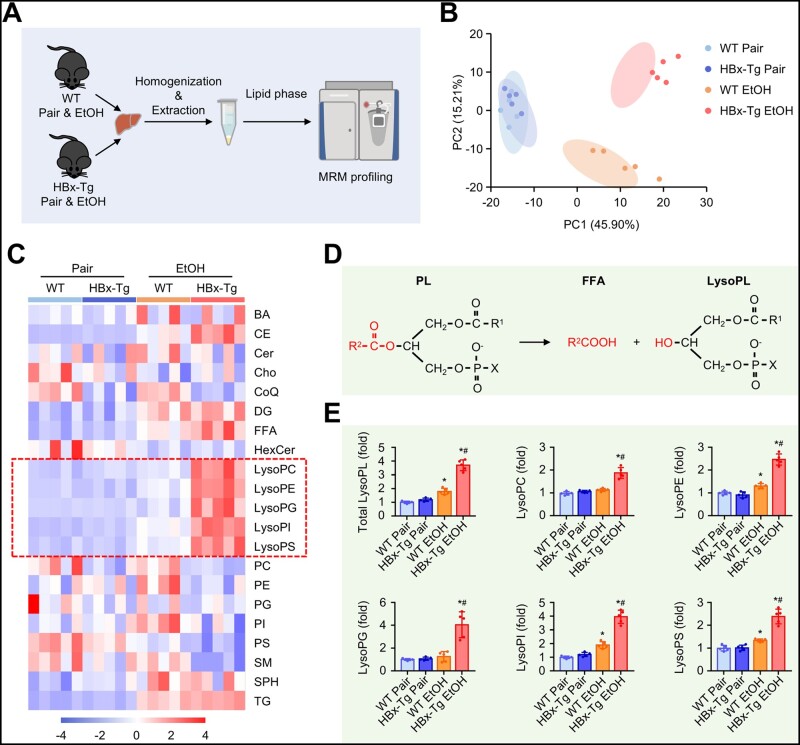
HBx upregulates ethanol-induced lysophospholipids production. (A) Schematic diagram of the lipidomics of livers from WT and HBx-Tg mice fed a control diet or ethanol diet. (B) Score scatter plot corresponding to a principal component analysis (PCA) of the lipidomic data. Each individual is represented by a dot. (C) Heat map showing all lipid species identified in the livers of the indicated groups. (D) Schematic diagram of lysophospholipids synthesis. (E) Relative levels of total lysophospholipids, lysoPC, lysoPE, lysoPG, lysoPI, and lysoPS in the livers of the indicated groups. Data are shown as the mean ± SD (*n *=* *5 in each group). ^*^P < 0.05 compared with the respective pair-fed group; ^#^*P* < 0.05 compared with the WT ethanol-fed group by one-way ANOVA with Tukey’s multiple comparison post-test. MRM, multiple reaction monitoring; BA, bile acid; CE, cholesteryl ester; Cer, ceramide; Cho, cholesterol; CoQ, coenzyme Q; DG, diglyceride; FFA, free fatty acid; HexCer, hexosyl-ceramide; lysoPC, lysophosphatidylcholine; lysoPE, lysophosphatidylethanolamine; lysoPG, lysophosphatidylglycerol; lysoPI, lysophosphatidylinositol; lysoPS, lysophosphatidylserine; PC, phosphatidylcholine; PE, phosphatidylethanolamine; PG, phosphatidylglycerol; PI, phosphatidylinositol; PS, phosphatidylserine; SM, sphingomyelin; SPH, sphingosine; TG, triacylglycerols.

To investigate the changes in the hepatic lipidome, we performed lipidomic analysis of livers from WT and HBx-Tg mice (Figure 2A). In total, we identified 585 lipid species in 21 lipid subclasses in the liver (Supplementary Figure S2A). Non-supervised principal component analysis of the lipidomic data demonstrated that the different metabolic clusters were associated with diet and mouse genotype (Figure 2B). Next, we used a heat map to display the global profile of 21 lipid classes in each group (Figure 2C). As expected, the levels of diglyceride (DG), triacylglycerols (TG), and cholesteryl ester (CE) were upregulated by ethanol consumption in WT mice and further increased in HBx-WT mice, except DG (Supplementary Figure S2B and C). Generally, as shown in Figure 2D, phospholipids (PLs) are hydrolysed by the enzymatic and chemical processes of phospholipases to release lysoPLs and free fatty acid (FFA) [29]. LysoPLs such as lysophosphatidylethanolamine (lysoPE), lysophosphatidylinositol (lysoPI), lysophosphatidylcholine (lysoPC), lysophosphatidylglycerol (lysoPG), and lysophosphatidylserine (lysoPS) have pronounced effects on diverse diseases, and the pro-inflammatory effects induced by these compounds have already been reviewed [19]. Interestingly, we observed that HBx strikingly increased the levels of lysoPLs (Figure 2E) and decreased the corresponding PLs (Supplementary Figure S2D), which were slightly changed or unchanged in WT mice after ethanol consumption. The levels of total FFA and arachidonic acid (an omega-6-polyunsaturated fatty acid that serves as substrate precursor for inflammatory lipid mediators) [30] were significantly increased in ethanol-fed HBx-Tg mice compared with ethanol-fed WT mice (Supplementary Figure S2E and F). Overall, ethanol-induced hepatic lipidome changes mostly in the TG, DG, and CE subclasses in WT and HBx-Tg mice, while HBx induced worse lipid profiles with high levels of lysoPLs (Supplementary Figure S3).

### HBx exacerbates ethanol-induced hepatic oxidative stress and lipid peroxidation in mice

**Figure 3.**
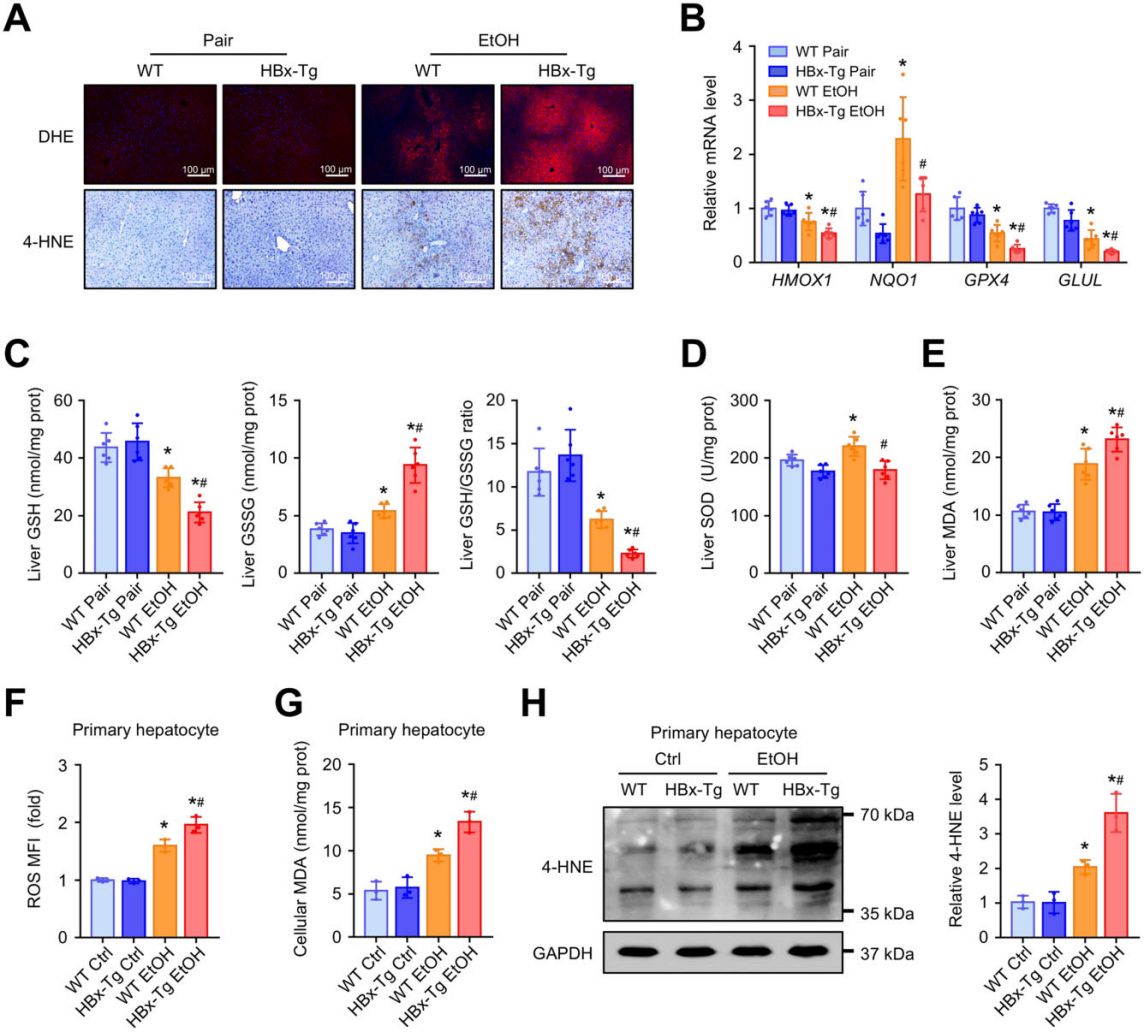
HBx exacerbates ethanol-induced hepatic oxidative stress and lipid peroxidation. (A) Representative DHE staining and immunohistochemical staining of 4-HNE (lower) of the liver sections. (B) Relative mRNA levels of genes related to the antioxidant response in the livers of mice in the indicated groups. (C) Liver GSH, GSSG, and GSH/GSSG ratios of mice in the indicated groups. (D) Liver SOD activity of mice in the indicated groups. (E) Liver MDA levels of mice in the indicated groups. (F)–(H) Primary hepatocytes isolated from WT and HBx-Tg mice were treated with 100 mM of ethanol for 24 h. (F) Relative cellular ROS detected by DCFH-DA and measured by using a multifunction micro reader. (G) Cellular MDA levels in the indicated groups. (H) Representative Western blot images showing 4-HNE protein levels followed by semi-quantitative analyses. GAPDH served as a loading control. Data are shown as the mean ± SD (*n *=* *3 or 6 in each group). For (B)–(E), ^*^P <* *0.05 compared with the respective pair-fed group; ^#^*P* < 0.05 compared with the WT ethanol-fed group by one-way ANOVA with Tukey’s multiple comparison post-test. For (F)–(H), ^*^P < 0.05 compared with the respective control-treated group; ^#^*P* < 0.05 compared with the WT ethanol-treated group by one-way ANOVA with Tukey’s multiple comparison post-test. 4-HNE, 4-hydroxynonenal; GSH, glutathione; GSSG, oxidative glutathione; SOD, superoxide dismutase; ROS, reactive oxygen species; MDA, malonaldehyde.

Perturbed lipid homeostasis results in steatosis, lipid peroxidation, inflammation, and fibrotic processes that are important pathophysiological determinants in ALD pathogenesis [17]. Dihydroethidium (DHE) staining results showed increased production of ROS after ethanol treatment, whereas HBx overexpression further increased ROS generation (Figure 3A, upper). In addition, 4-hydroxynonenal (4-HNE), an end product of lipid peroxidation, was elevated in both ethanol-fed WT and HBx-Tg mice, with a greater increase in HBx-Tg mice (Figure 3A, lower). We further measured several ROS-related genes, such as *Hmox1*, *Nqo1*, *Gpx4*, and *Glul*. As shown in Figure 3B, the levels of the above genes were markedly decreased in ethanol-fed HBx-Tg mice compared with ethanol-fed WT mice. Accordingly, the contents of GSSG (Figure 3C) and MDA (Figure 3E) were markedly elevated in ethanol-fed HBx-Tg mice, while the GSH content, GSH/GSSG ratio (Figure 3C), and superoxide dismutase (SOD) activity (Figure 3D) were significantly decreased in ethanol-fed HBx-Tg mice compared with those in WT mice. In *in vitro* experiments, we observed that ethanol treatment markedly increased ROS production in primary hepatocytes isolated from WT and HBx-Tg mice, with higher ROS levels in primary hepatocytes from HBx-Tg mice (Figure 3F). The cellular MDA content was also higher in primary hepatocytes from HBx-Tg mice than in those from WT mice after ethanol treatment (Figure 3G). 4-HNE adducts in primary hepatocytes were detected by Western blotting. As expected, the level of 4-HNE in ethanol-treated primary hepatocytes from HBx-Tg mice was significantly increased compared with that in hepatocytes from WT mice (Figure 3H). Overall, these findings indicate that HBx aggravates ethanol-induced oxidative stress and lipid peroxidation *in vivo* and *in vitro*.

### HBx increases acetaldehyde accumulation in ethanol-fed mice

**Figure 4.**
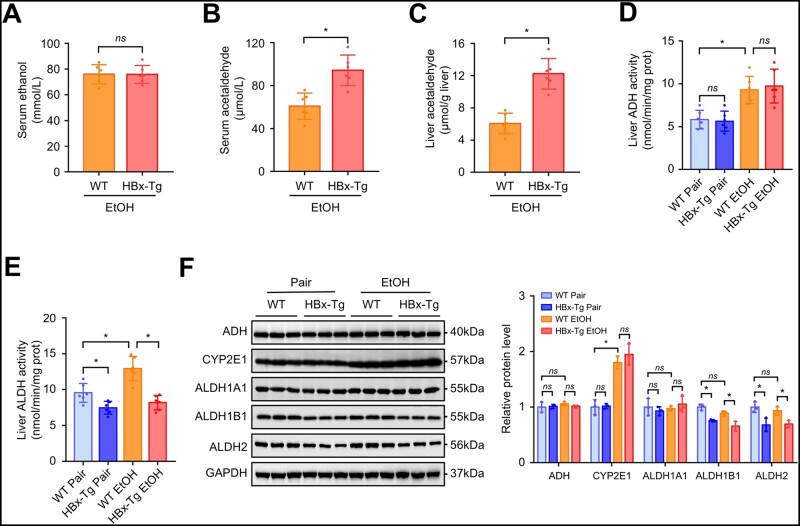
HBx increases acetaldehyde accumulation in mice. (A) Serum ethanol concentrations of mice in the indicated groups. (B) Serum acetaldehyde concentrations of mice in the indicated groups. (C) Liver acetaldehyde concentrations of mice in the indicated groups. (D) Liver ADH activity of mice in the indicated groups. (E) Liver ALDH activity of mice in the indicated groups. (F) Western blot images showing the protein levels of hepatic ethanol and acetaldehyde enzymes followed by semi-quantitative analyses. GAPDH served as a loading control. Data are shown as the mean ± SD (*n *=* *3 or 6 in each group). For (A)–(C), *ns*, not significant, ^*^P < 0.05 by two-tailed Student’s *t*-test. For (D)–(F), ^*^P < 0.05 by one-way ANOVA with Tukey’s multiple comparison post-test. ADH, alcohol dehydrogenase; ALDH, acetaldehyde dehydrogenase.

As ROS mainly originate from the process of ethanol metabolism in ALD, we questioned whether HBx could alter ethanol metabolism and subsequently affect the susceptibility to ethanol-induced steatohepatitis. However, there was no difference in the level of serum ethanol between WT and HBx-Tg mice (Figure 4A). Interestingly, the level of serum acetaldehyde in ethanol-fed HBx-Tg mice was higher than that in ethanol-fed WT mice (Figure 4B). Moreover, the level of liver acetaldehyde was also higher in HBx-Tg mice than in WT mice (Figure 4C). To determine the mechanism of acetaldehyde accumulation, we examined liver ADH activity and liver ALDH activity. We found that HBx had no impact on liver ADH activity (Figure 4D). Chronic-binge ethanol consumption increased liver ALDH activity in WT mice—an effect that was blocked in HBx-Tg mice (Figure 4E). Moreover, basal liver ALDH activity was lower in HBx-Tg mice than in WT mice. Furthermore, we examined several enzymes related to ethanol metabolism. HBx did not affect the protein levels of ADH and CYP2E1 (Figure 4F), which are responsible for metabolizing ethanol into acetaldehyde. However, HBx significantly decreased the protein level of ALDH2, which is mainly responsible for detoxifying acetaldehyde. ALDH1B1, another mitochondrial ALDH isoenzyme, was also reduced in HBx-Tg mice compared with WT mice, while ALDH1A1, a cytosolic ALDH isoenzyme, was not affected by HBx. These results indicate that HBx inhibits acetaldehyde clearance in ethanol-fed mice.

### Acetaldehyde induces lysophospholipids generation in an ROS-dependent manner *in vitro*

**Figure 5.**
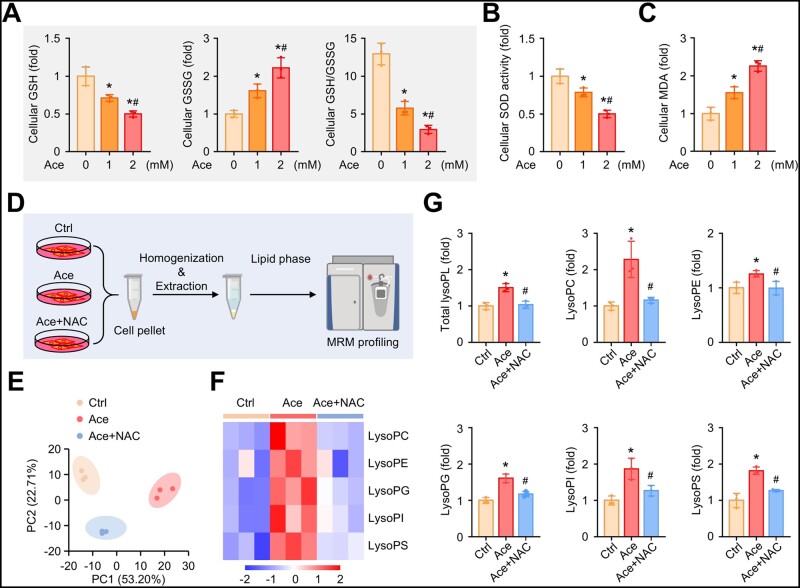
Acetaldehyde induces lysophospholipids generation in an ROS-dependent manner. (A)–(C) LO2 cells were treated with different concentrations of acetaldehyde (1 and 2 mM) for 48 h. (A) Relative GSH and GSSG levels and GSH/GSSG ratios of cells in the indicated groups. (B) Relative SOD activity levels of cells in the indicated groups. (C) Relative MDA levels of cells in the indicated groups. (D)–(G) LO2 cells were treated with 2 mM of acetaldehyde in the absence or presence of 10 mM of NAC for 48 h. (D) Schematic diagram of the lipidomics of cells. (E) Score scatter plot corresponding to a principal component analysis (PCA) of the lipidomic data. Each individual is represented by one dot. (F) Heat map showing lysophospholipid species identified in the cells of the indicated groups. (G) Relative levels of total lysophospholipids, lysoPC, lysoPE, lysoPG, lysoPI, and lysoPS in the cells of the indicated groups. Data are shown as the mean ± SD (*n *=* *3 in each group). For (A)–(C), ^*^P < 0.05 compared with the control group; ^#^*P* < 0.05 compared with the 1 mM of Ace-treated group by one-way ANOVA with Tukey’s multiple comparison post-test. For (G), ^*^P < 0.05 compared with the control group; ^#^*P* < 0.05 compared with the 2 mM of Ace-treated group by one-way ANOVA with Tukey’s multiple comparison post-test. Ace, acetaldehyde; GSH, glutathione; GSSG, oxidative glutathione; SOD, superoxide dismutase; MDA, malonaldehyde; NAC, N-Acetylcysteine.

As oxidative stress has been implicated in lysophospholipids generation [31] and acetaldehyde-induced cytotoxicity [32], to understand the relationship between elevated acetaldehyde and aberrant lysophospholipids synthesis in ethanol-fed HBx-Tg mice, we treated LO2 cells (a normal human hepatocyte cell line) with acetaldehyde (acetaldehyde was used at 1 mM and 2 mM to induce oxidative stress [32, 33]). Acetaldehyde caused reductions in GSH content, GSH/GSSG ratio (Figure 5A), and SOD activity (Figure 5B) in a dose-dependent manner. In contrast, the levels of GSSG and MDA were significantly increased in LO2 cells treated with acetaldehyde in comparison with those of the control cells (Figure 5A and C). Furthermore, we treated LO2 cells with 2 mM of acetaldehyde in the presence of 10 mM of NAC, a well-known antioxidant, and performed lipidomic analysis of LO2 cells to determine whether there was a shift in the lipidome after acetaldehyde treatment (Figure 5D). The lipid composition of LO2 cells treated with acetaldehyde showed significant differences compared with control cells (Figure 5E). In total, we identified 21 lipid classes and 431 lipid species in LO2 cells (Supplementary Figure S4A). Consistently with the lipidome of the *in vivo* study, lysoPLs were significantly more abundant in acetaldehyde-treated LO2 cells and NAC significantly attenuated the elevation of lysoPLs production induced by acetaldehyde (Figure 5F and G, and Supplementary Figure S4B–E). Taken together, these results suggest that acetaldehyde induces lysophospholipid generation in an ROS-dependent manner.

### HBx suppresses ALDH activity by decreasing mitochondrial ALDH2 in hepatocytes

**Figure 6.**
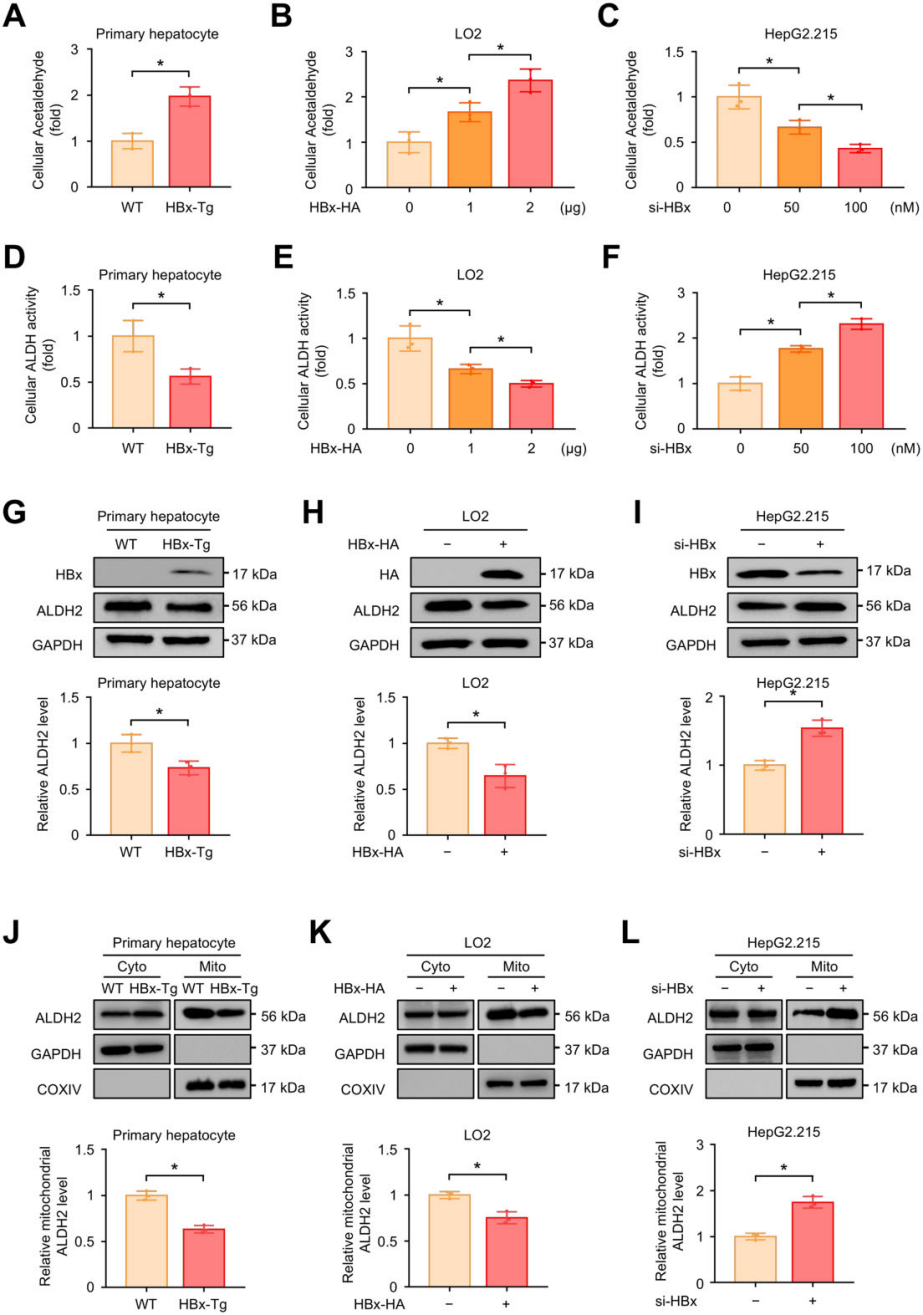
HBx suppresses ALDH activity by decreasing mitochondrial ALDH2 in hepatocytes. (A) Primary hepatocytes isolated from WT and HBx-Tg mice were treated with 2 mM of acetaldehyde for 2 h. Relative acetaldehyde levels of cells were analysed. (B) LO2 cells were transfected with the indicated concentrations of HBx-HA plasmids. Forty-eight hours after transfection, LO2 cells were treated with 2 mM of acetaldehyde for 2 h. Relative acetaldehyde levels of cells were analysed. (C) HepG2.215 cells were transfected with the indicated concentrations of siRNA for HBx. Forty-eight hours after transfection, HepG2.215 cells were treated with 2 mM of acetaldehyde for 2 h. Relative acetaldehyde levels of cells were analysed. (D)–(F) Relative cellular ALDH activity of primary hepatocytes (D), LO2 cells (E), and HepG2.215 cells (F) of the indicated group. (G)–(I) Representative Western blot images showing the indicated proteins followed by semi-quantitative analyses in primary hepatocytes (G), LO2 cells (H), and HepG2.215 cells (I) of the indicated groups. GAPDH served as a loading control. (J)–(L) Representative Western blot images of ALDH2 followed by semi-quantitative analyses in the cytosolic/mitochondrial fractions of primary hepatocytes (J), LO2 cells (K), and HepG2.215 cells (L) of the indicated groups, respectively. GAPDH and COXIV served as cytosolic and mitochondrial controls, respectively. Data are shown as the mean ± SD (*n *=* *3 in each group). For (A), (D), and (G)–(L), ^*^P < 0.05 by two-tailed Student’s *t*-test. For (B), (C), (E), and (F), ^*^P < 0.05 by one-way ANOVA with Tukey’s multiple comparison post-test.

We next examined the levels of acetaldehyde accumulation in cells after acetaldehyde stimuli to further investigate the inhibitory role of HBx on hepatocyte ALDH activity. As shown in Figure 6A–C, the presence of HBx delayed acetaldehyde clearance in hepatocytes. Correspondingly, cellular ALDH activity was decreased in HBx-expressing hepatocytes (Figure 6D–F). As ALDH2 is the most potent enzyme responsible for acetaldehyde clearance, we then examined the expression of ALDH2 in whole cell lysates, cytosolic fractions, and mitochondrial fractions. Consistently with the results of the *in vivo* study, we found that HBx overexpression decreased the total ALDH2 level in primary hepatocytes and LO2 cells (Figure 6G and H). Knock-down of HBx by siRNA increased ALDH2 expression in HepG2.215 cells (Figure 6I). Interestingly, we found that the reduction in ALDH2 caused by HBx overexpression mainly occurred in mitochondrial fractions and not cytosolic fractions (Figure 6J and K). Knock-down of HBx also increased ALDH2 expression in the mitochondrial fractions of HepG2.215 cells (Figure 6L). To confirm that the decrease in mitochondrial ALDH2 by HBx is responsible for acetaldehyde accumulation, we overexpressed ALDH2 by plasmids in HBx-overexpressing LO2 cells (Supplementary Figure S5A). We found that ALDH2 overexpression rescued the decreased ALDH activity in HBx-overexpressing LO2 cells (Supplementary Figure S5B) and promoted acetaldehyde clearance after acetaldehyde treatment (Supplementary Figure S5C). Overall, these results demonstrate that HBx suppresses ALDH activity by decreasing mitochondrial ALDH2 in hepatocytes.

### HBx interacts with mitochondrial ALDH2

**Figure 7.**
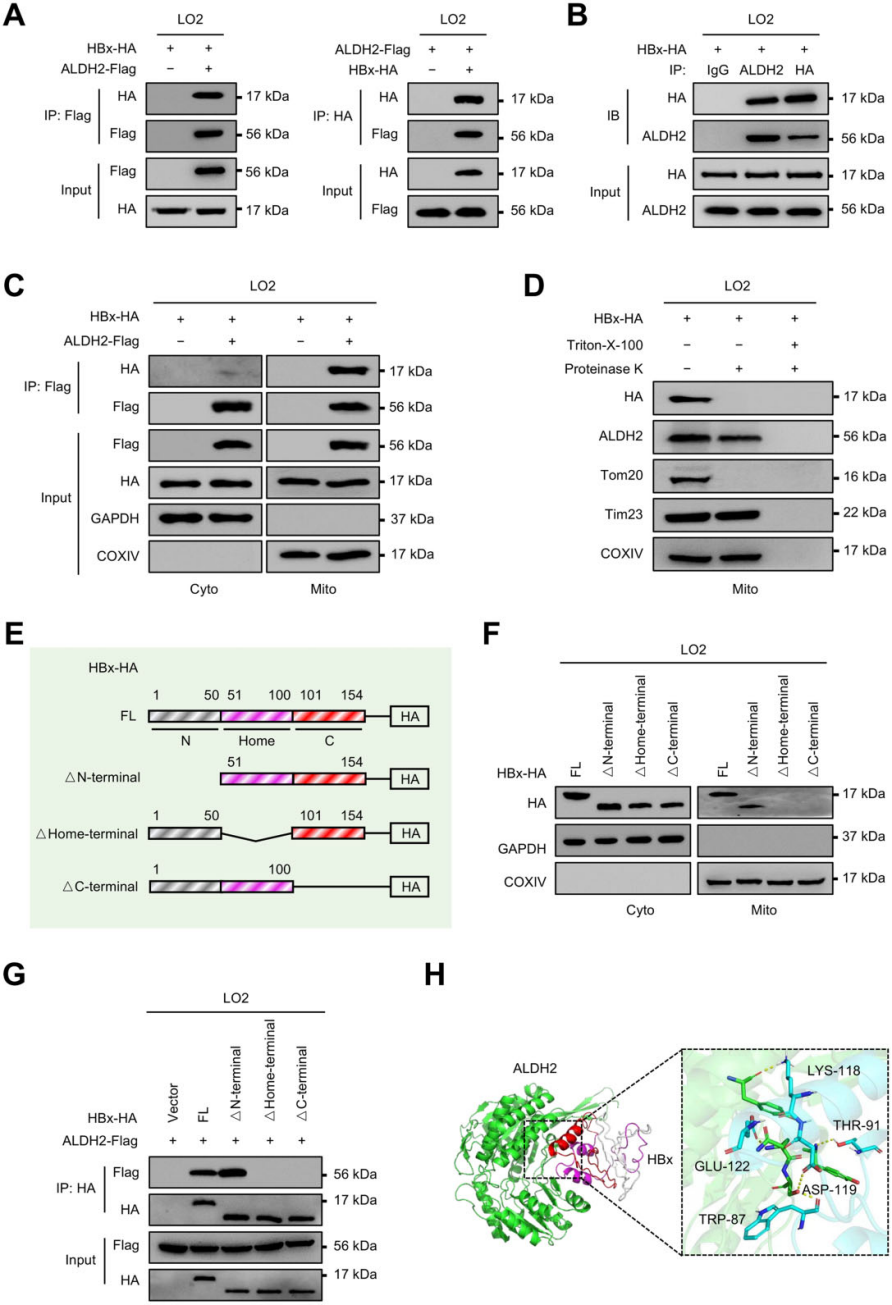
HBx interacts with mitochondrial ALDH2. (A) Co-IP assays to evaluate the interaction of HBx and exogenous ALDH2 in LO2 cells transfected with HBx-HA and ALDH2-Flag plasmids. (B) Co-IP assays confirmed the interaction of HBx and endogenous ALDH2 in LO2 cells transfected with HBx-HA plasmid. (C) The cytosolic and mitochondrial fractions from LO2 cells transfected with HBx-HA and ALDH2-Flag plasmids were subjected to immunoprecipitation with anti-Flag antibody, and the immunoprecipitated proteins were immunoblotted with the indicated antibodies. GAPDH and COXIV served as cytosolic and mitochondrial controls, respectively. (D) Mitochondria from LO2 cells transfected with HBx-HA plasmid were fractionated and incubated with (Lanes 2 and 3) or without (Lane 1) proteinase K. To disrupt mitochondrial integrity, Triton X-100 was added along with proteinase K (Lane 3). The expression of HBx-HA was detected by using immunoblotting. Tomm20 (outer membrane), Timm23 (inner membrane), COXIV (inner membrane), and ALDH2 (matrix) were used as submitochondrial fraction markers. (E) Schematic diagram of HBx-HA and its truncation mutants. (F) The cytosolic and mitochondrial fractions from LO2 cells transfected with the indicated expression plasmids. The fractionated proteins were immunoblotted with the indicated antibodies. GAPDH and COXIV served as cytosolic and mitochondrial controls, respectively. (G) Co-IP assays to confirm the interaction of HBx domains with ALDH2. (H) Molecular docking simulation between HBx and ALDH2.

To reveal the potential mechanism by which HBx decreased mitochondrial ALDH2, we next constructed Flag-tagged ALDH2-overexpressing and HA-tagged HBx-overexpressing LO2 cells to examine the interaction between ALDH2 and HBx. Further co-immunoprecipitation (co-IP) assays were carried out and revealed that exogenous ALDH2 strongly interacted with HBx (Figure 7A). We next confirmed the interaction between endogenous ALDH2 and HBx via co-IP assays (Figure 7B). As ALDH2 is mainly localized in the mitochondria, we then separated the cytosolic and mitochondrial fractions of LO2 cells using density-gradient centrifugation methods. The two separated fractions were subjected to co-IP assays and immunoblot analyses. Interestingly, mitochondrial ALDH2 strongly immunoprecipitated with HBx, while the interaction between cytosolic ALDH2 and HBx was very weak (Figure 7C). In previous studies [34, 35], mitochondrial HBx was mainly located in the outer mitochondrial membrane (OMM). Proteins located in the OMM are expected to be protease-sensitive, while internal proteins are digested only after disruption of the mitochondrial membrane by Triton X-100 [36]. Immunoblot analyses showed that ALDH2 (a mitochondrial matrix protein), Tim23 (an inner mitochondrial membrane protein), and COXIV (an inner mitochondrial membrane protein) were resistant to proteinase K, whereas HBx and Tom20 (an OMM protein) were degraded. When the mitochondrial fractions were treated with proteinase K in the presence of Triton X-100, all tested proteins were degraded (Figure 7D). The mitochondrial protein-localization assay suggested that HBx mainly interacted with ALDH2 in the OMM.

To search for the critical amino acid residues in HBx responsible for its interaction with ALDH2, we divided HBx into three domains and established three truncation mutants (Figure 7E). We found that only the full-length HBx protein (1–154 aa) and its truncation mutant HBx (51–154 aa, lacking the N-terminus) were able to localize to mitochondria. In contrast, the other two truncation mutants HBx (1–50 aa/101–154 aa, lacking the Home-terminus) and HBx (1–100 aa, lacking the C-terminus) failed to localize to mitochondria (Figure 7F). Further co-IP assays verified that HBx (1–50 aa/101–154 aa, lacking the Home-terminus) and HBx (1–100 aa, lacking the C-terminus) were unable to interact with ALDH2 (Figure 7G). Next, we performed a molecular docking simulation using the program PYMOL and found the 87, 91, 118, 119, and 122 aa of HBx are the positively charged residues that bind to ALDH2 (Figure 7H). The structure of ALDH2 was obtained from a pdb file (accession number: 1cw3) and the structure of HBx was predicted using the AlphaFold2 model. These results suggest that HBx may exert its inhibitory function by interacting with mitochondrial ALDH2 and the Home-terminal and C-terminal domains of HBx are responsible for its mitochondrial localization and interaction with ALDH2.

### HBx promotes degradation of mitochondrial ALDH2 via the ubiquitin–proteasome pathway

**Figure 8.**
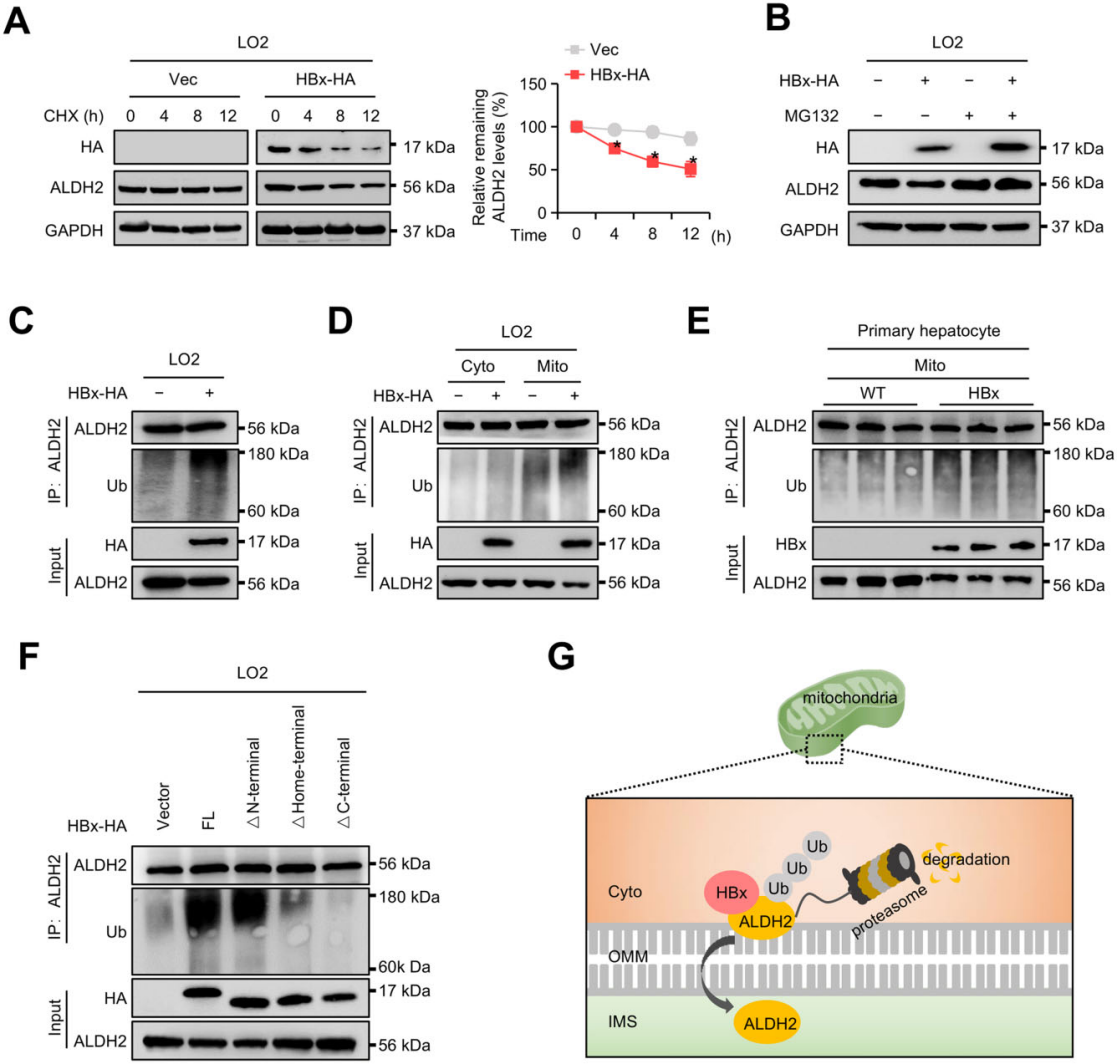
HBx promotes degradation of mitochondrial ALDH2 via the ubiquitin–proteasome pathway. (A) Representative Western blot images showing the ALDH2 protein levels in LO2 cells transfected with HBx-HA or empty vector. The transfected cells were treated with 100 μg/mL of CHX for the indicated time periods. GAPDH served as a loading control. (B) Representative Western blot images showing the ALDH2 protein levels in LO2 cells transfected with HBx-HA or empty vector. The transfected cells were treated with 20 μM of MG132 for 6 h before being harvested. GAPDH served as a loading control. (C) Representative Western blot images showing the ubiquitination of ALDH2 in LO2 cells transfected with HBx-HA or empty vector. (D) Representative Western blot images showing the ubiquitination of ALDH2 in the cytosolic and mitochondrial fractions of LO2 cells transfected with HBx-HA or empty vector. (E) Western blot images showing the ubiquitination of ALDH2 in the mitochondrial fractions of primary hepatocytes from WT and HBx-Tg mice. (F) Representative Western blot images showing the ubiquitination of ALDH2 in LO2 cells transfected with the indicated expression plasmids. (G) Schematic diagram of ubiquitin-dependent degradation of ALDH2. ALDH2 interacts with HBx in the outer mitochondrial membrane, leading to degradation by the proteasome. Data in (A) are shown as the mean ± SD (*n *=* *3 in each group). ^*^P < 0.05 by two-way ANOVA with Bonferroni’s multiple comparison post-test. Cyto, cytoplasm; OMM, outer mitochondrial membrane; IMS, intermembrane space of mitochondria.

It has been well established that the mistargeting of mitochondrial proteins triggers multiple degradation pathways [37, 38]. We therefore postulated that the interaction between HBx and ALDH2 in the OMM may promote ALDH2 degradation. CHX chasing assays revealed that HBx shortened the half-life of endogenous ALDH2 (Figure 8A). A previous study demonstrated that ALDH2 can be degraded by ubiquitination [39]. In our study, the addition of MG132, a 26S proteasome inhibitor, completely abolished HBx-induced degradation of ALDH2 (Figure 8B). Consistently with this finding, the ubiquitination of ALDH2 was significantly increased by HBx overexpression (Figure 8C). Furthermore, we separated the cytosolic and mitochondrial fractions of LO2 cells transfected with HBx-HA or vector plasmids. The two separated fractions were subjected to analysis of the ubiquitination of ALDH2. Notably, HBx mainly promoted the ubiquitination of mitochondrial ALDH2 and not cytosolic ALDH2 (Figure 8D). Similar results were obtained in primary hepatocytes isolated from WT and HBx-Tg mice. HBx remarkably promoted the ubiquitination of mitochondrial ALDH2 (Figure 8E). In addition, both the truncation mutants HBx (1–50 aa/101–154 aa, lacking the Home-terminus) and HBx (1–100 aa, lacking the C-terminus), which were unable to interact with ALDH2, lost the ability to promote the ubiquitination of ALDH2 (Figure 8F). A schematic diagram of the ubiquitin-dependent degradation of ALDH2 is shown in Figure 8G. Overall, these data suggest that HBx promotes mitochondrial ALDH2 degradation in a ubiquitin-dependent manner.

### ALDH2 is reduced in HBV-infected human liver and interacted with HBx

**Figure 9.**
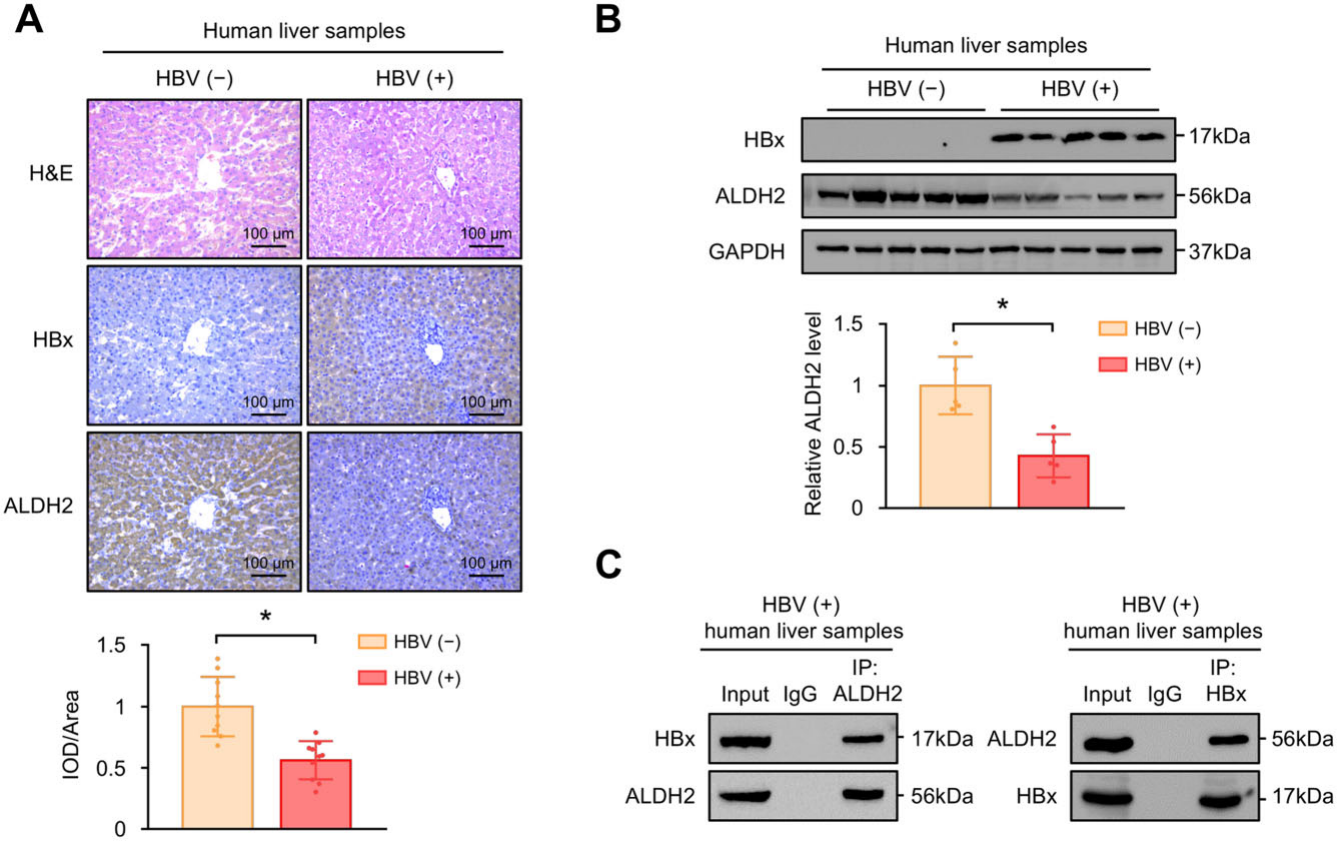
ALDH2 is reduced in HBV-infected human liver. (A) Representative immunohistochemical staining of HBx and ALDH2 in non-HBV-infected (*n *=* *10) and HBV-infected (*n *=* *10) human livers. Quantification of ALDH2 protein expression levels in liver sections based on IHC staining results by using Image J. (B) Western blot images showing ALDH2 and HBx protein levels followed by semi-quantitative analyses in non-HBV-infected (*n *=* *5) and HBV-infected (*n *=* *5) human livers. GAPDH served as a loading control. (C) Co-IP assays to evaluate the interaction of HBx and ALDH2 in HBV-infected human liver. Data are shown as the mean ± SD. ^*^P < 0.05 by two-tailed Student’s *t*-test.

To extend our findings to the pathophysiological state in human subjects, we further examined the expression levels of ALDH2 in human liver samples. Consistently with lower ALDH2 protein levels in HBx-Tg mice, immunohistochemical staining revealed that ALDH2 was markedly reduced in hepatocytes from individuals with HBV infection (Figure 9A). In addition, Western blot also confirmed the downregulation of ALDH2 in HBV-infected liver (Figure 9B). Furthermore, the endogenous interaction of HBx and ALDH2 was confirmed by co-IP assays (Figure 9C). To further mimic HBV infection *in vitro*, we employed HepG2-NTCP cells with stable overexpression of the HBV receptor (Supplementary Figure S6A and B) and confirmed the downregulation of ALDH2 after HBV infection (Supplementary Figure S6C). In addition, the ubiquitination levels of ALDH2 were also increased in HBV-infected HepG2-NTCP cells (Supplementary Figure S6D). Collectively, these data confirm the interaction between ALDH2 and HBx in human subjects and imply that ALDH2 is reduced in HBV-infected human liver.

## Discussion

In the current study, we demonstrated that HBx aggravated ethanol-induced steatohepatitis by using a HBx-Tg mice model. Mechanistically, we demonstrated that HBx directly bound to ALDH2 in the OMM and promoted its ubiquitination and degradation. When HBx was overexpressed via gene transfer in mice or plasmids in cells, mitochondrial ALDH2 was decreased along with reduced ALDH activity, leading to acetaldehyde accumulation. Furthermore, acetaldehyde induced lipid peroxidation and lysophospholipids production to aggravate alcoholic steatohepatitis. A schematic diagram of the interaction between HBx and mitochondrial ALDH2 is shown in the Graphical Abstract.

Epidemiologic investigations show that chronic alcoholics with HBV infection develop chronic liver disease and hepatocellular carcinoma at an earlier age than alcoholics without HBV infection [40], which suggests synergy between alcohol and HBV in liver disease development. Increased HBV replication in response to ethanol has been reported in humans, mice, and cells, and several mechanisms have been proposed for the regulation of HBV replication [41–43]. In the present study, we also confirmed that ethanol could promote HBV replication and upregulate the expression of HBx. To mimic virus-triggered HBx expression as found in patients with HBV infection, we used the HBx-Tg mouse model and demonstrated that mice with hepatic HBx expression were more sensitive to ethanol-induced steatohepatitis due to the downregulation of mitochondrial ALDH2.

Excessive lipid accumulation leads to lipotoxicity, which is the predominant hallmark of ALD [44]. In our study, we found that HBx impacted the hepatic lipidome of mice fed an ethanol diet by using lipidomic analysis. Notably, the most obvious difference we observed was the elevation of lysoPLs. The levels of lysoPLs, a class of bioactive lipid mediators, have been reported to be elevated in diverse diseases, including ALD [18–20]. Most lysoPLs, especially lysoPC, are known to exhibit pro-inflammatory properties and may be generated by two different pathways: either by the action of phospholipase A2 or by ROS that are generated in significant amounts under inflammatory conditions [31]. In the present study, we found that acetaldehyde levels, but not ethanol levels, were markedly elevated in both the serum and liver of ethanol-fed HBx-Tg mice compared with those of ethanol-fed WT mice, suggesting that HBx inhibited the activity of acetaldehyde clearance. Acetaldehyde has been considered a culprit in the development of ALD given its role in forming a variety of proteins and DNA adducts to impair cellular function, deplete mitochondrial glutathione, and promote oxidative stress and lipid peroxidation [14]. Interestingly, the acetaldehyde-treated cells had many similar features to those observed in ethanol-fed HBx-Tg mouse liver, such as elevated lysophospholipids. However, the above changes were partly rescued by the ROS inhibitor NAC, suggesting that acetaldehyde likely increased the levels of lysophospholipids in an ROS-dependent manner. We speculated that acetaldehyde induced oxidative stress and lipid peroxidation to generate oxidized phospholipids, which provided substrates for phospholipases. In addition, acetaldehyde has been reported to elevate the intracellular Ca^2+^ level [32], which may activate Ca^2+^-dependent phospholipases, such as Ca^2+^-dependent cytosolic phospholipases. The precise mechanism by which acetaldehyde induces lysophospholipids production will be elucidated in our future study.

Acetaldehyde is mainly generated in the liver from ethanol by ADH after absorption and subsequently detoxified by ALDH family members. Previous study has reported that the polymorphism of ALDH2 rs671 may play a hazardous role in HBV infection and persistence [45]. However, little is known about the potential role of HBx as a mediator of acetaldehyde metabolism in alcoholic steatohepatitis. Among ALDH family members, ALDH2 is the most potent isoenzyme responsible for acetaldehyde detoxification. The robust capacity of ALDH2 for aldehyde detoxification renders it a promising target for the treatment of ALD [46, 47]. In the present study, we found that ALDH2 was significantly reduced in the HBV-infected human liver as well as HBx-Tg mice. co-IP assays confirmed the interaction between HBx and ALDH2, and the OMM was the main cellular compartment in which HBx–ALDH2 interaction occurred. It was reported that 54–70, 75–88, and 109–131 aa were the critical residues of HBx required for mitochondrial localization [48]. In this study, we confirmed that 51–100 and 101–154 aa of HBx were required for its mitochondrial localization and interaction with ALDH2. It has been reported that mitochondrial proteins will be ubiquitinated and degraded by the proteasome in the case of failure to transport to the correct submitochondrial compartment [49]. A previous study revealed that ALDH2 stability could be regulated by the ubiquitin–proteasome pathway [39]. One of our hypotheses is that the interaction between HBx and ALDH2 clogs the transporting channel in the OMM, inhibiting ALDH2 translocation to the mitochondrial matrix. Mistargeted ALDH2 may trigger the monitoring mechanisms that remove arrested mitochondrial proteins [37, 38].

However, several limitations should be noted in our work. First, although HBx-Tg mice are widely used for studying HBV-related human disease, our experimental model still could not totally mimic the status of HBV infection in clinical patients and an HBV-infected model is needed to confirm our conclusion in the future. Second, the detailed regulatory mechanisms by which HBx regulates the ubiquitination of mitochondrial ALDH2 are still unclear and need to be further investigated. Third, the sample size for human study in this study is limited. We will validate our mouse data in a large clinical sample with different stages of HBV infection and different alcohol exposure conditions in our further research, which will help to improve the translational potential of this work.

## Conclusions

We demonstrated that ALDH2 was downregulated in HBV-infected livers and that HBx induced ALDH2 ubiquitin-dependent degradation to enhance alcoholic steatohepatitis. Our study provided a novel explanation for the sensitization of HBV-infected patients to alcohol-induced liver disease and it should be worth determining whether targeting the HBx–ALDH2 interaction can be a promising pharmacological therapeutic strategy for ALD with HBV infection.

## Supplementary Material

goad006_Supplementary_Data
