# Supplementary material for: Hepatitis B virus X protein induces ALDH2 ubiquitin-dependent degradation to enhance alcoholic steatohepatitis
Source: Gastroenterol Rep (Oxf). 2023 Mar 1;11:goad006. doi: 10.1093/gastro/goad006 (PMC9978578; doi:10.1093/gastro/goad006)
Supplement: goad006_Supplementary_Data [file goad006_supplementary_data.docx]

**Supplementary materials**

**Supplementary Table 1. Characteristics of human subjects in this study**

| No | Group | Age | Gender | HBV DNA (IU/ml) | HBV stage | Operation |
| --- | --- | --- | --- | --- | --- | --- |
| #01 | HBV (-) | 29 | Male | N/A | N/A | Partial hepatectomy |
| #02 | HBV (-) | 45 | Male | N/A | N/A | Partial hepatectomy |
| #03 | HBV (-) | 35 | Male | N/A | N/A | Partial hepatectomy |
| #04 | HBV (-) | 38 | Male | N/A | N/A | Partial hepatectomy |
| #05 | HBV (-) | 42 | Male | N/A | N/A | Partial hepatectomy |
| #06 | HBV (-) | 31 | Male | N/A | N/A | Partial hepatectomy |
| #07 | HBV (-) | 47 | Male | N/A | N/A | Partial hepatectomy |
| #08 | HBV (-) | 52 | Male | N/A | N/A | Partial hepatectomy |
| #09 | HBV (-) | 36 | Male | N/A | N/A | Partial hepatectomy |
| #10 | HBV (-) | 43 | Male | N/A | N/A | Partial hepatectomy |
| #11 | HBV (+) | 34 | Male | 4.09E+05 | Chronic hepatitis | Partial hepatectomy |
| #12 | HBV (+) | 32 | Male | 5.96E+04 | Chronic hepatitis | Partial hepatectomy |
| #13 | HBV (+) | 41 | Male | 5.58E+06 | Chronic hepatitis | Partial hepatectomy |
| #14 | HBV (+) | 49 | Male | 7.62E+03 | Chronic hepatitis | Partial hepatectomy |
| #15 | HBV (+) | 36 | Male | 3.84E+03 | Chronic hepatitis | Partial hepatectomy |
| #16 | HBV (+) | 43 | Male | 1.06E+04 | Chronic hepatitis | Liver biopsy |
| #17 | HBV (+) | 27 | Male | 8.57E+03 | Chronic hepatitis | Liver biopsy |
| #18 | HBV (+) | 32 | Male | 8.96E+03 | Chronic hepatitis | Liver biopsy |
| #19 | HBV (+) | 50 | Male | 1.15E+05 | Chronic hepatitis | Liver biopsy |
| #20 | HBV (+) | 48 | Male | 1.44E+04 | Chronic hepatitis | Liver biopsy |

N/A, not available.

**Supplementary Table 2. Primers for real-time PCR analysis**

| **Name** |  | **Sequence (5'-3')** | |
| --- | --- | --- | --- |
| HBV pgRNA | F | GCCTTAGAGTCTCCTGAGCA | |
|  | R | GAGGGAGTTCTTCTTCTAGG |  |
| HBx | F | ATGGCTGCTAGGCTGTGC | |
|  | R | TTAGGCAGAGGGGAAAAAGTTG | |
| Mouse IL1α | F | CACAACTGTTCGTGAGCGCT | |
|  | R | TTGGTGTTTCTGGCAACTCCT | |
| Mouse IL1β | F | GCAACTGTTCCTGAACTCAACT | |
|  | R | ATCTTTTGGGGTCCGTCAACT | |
| Mouse IL6 | F | AGGATACCACTCCCAACAGACCT | |
|  | R | CAAGTGCATCATCGTTGTTCATAC | |
| Mouse TNFα | F | TTCTGTCTACTGAACTTCGGGGTGATCGGTCC | |
|  | R | GTATGAGATAGCAAATCGGCTGACGGTGTGGG | |
| Mouse ICAM1 | F | GTGATGCTCAGGTATCCATCCA | |
|  | R | CACAGTTCTCAAAGCACAGCG | |
| Mouse VCAM1 | F | AGTTGGGGATTCGGTTGTTCT | |
|  | R | CCCCTCATTCCTTACCACCC | |
| Mouse HMOX1 | F | AAGCCGAGAATGCTGAGTTCA | |
|  | R | GCCGTGTAGATATGGTACAAGGA | |
| Mouse NQO1 | F | AGGATGGGAGGTACTCGAATC | |
|  | R | AGGCGTCCTTCCTTATATGCTA | |
| Mouse GPX4 | F | GCCTGGATAAGTACAGGGGTT | |
|  | R | CATGCAGATCGACTAGCTGAG | |
| Mouse GLUL | F | TGAACAAAGGCATCAAGCAAATG | |
|  | R | CAGTCCAGGGTACGGGTCTT | |


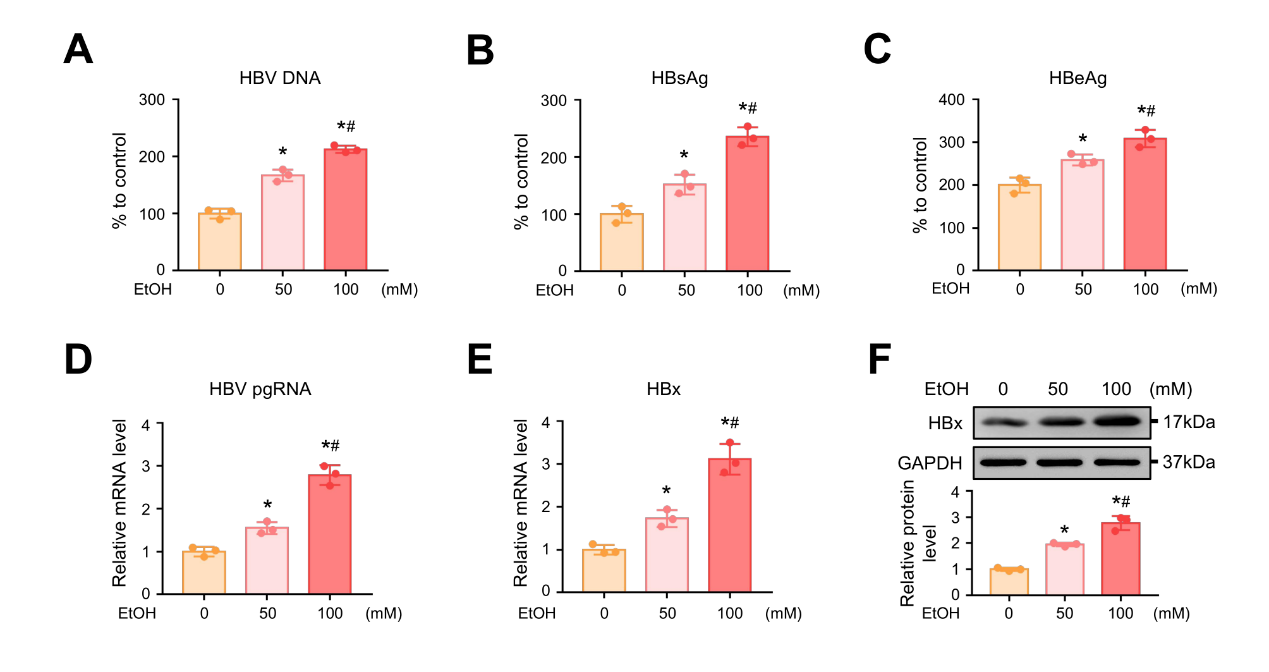


**Supplementary Figure 1. Ethanol promotes HBV replication and HBV gene transcription as well as HBx overexpression.**

**(A**-**F)** HepG2.215 cells were treated with different concentrations of ethanol (50 and 100 mM) for 48 hours. **(A)** Relative HBV DNA levels in the culture supernatants measured by the COBAS® TaqMan 48® assay. **(B** and **C)** Relative HBsAg and HBeAg levels in the culture supernatants measured by ELISA. **(D** and **E)** Relative mRNA levels of HBV pgRNA and HBx analyzed by real-time PCR. **(F)** Western blot images showing HBx protein levels followed by semi-quantitative analyses. GAPDH served as a loading control. Data are shown as the mean ± SD (*n* = 3 in each group). ^*^*P* < 0.05 compared with the control group; ^#^*P* < 0.05 compared with the 50 mM ethanol-treated group by one-way ANOVA with Tukey’s multiple comparison post-test.


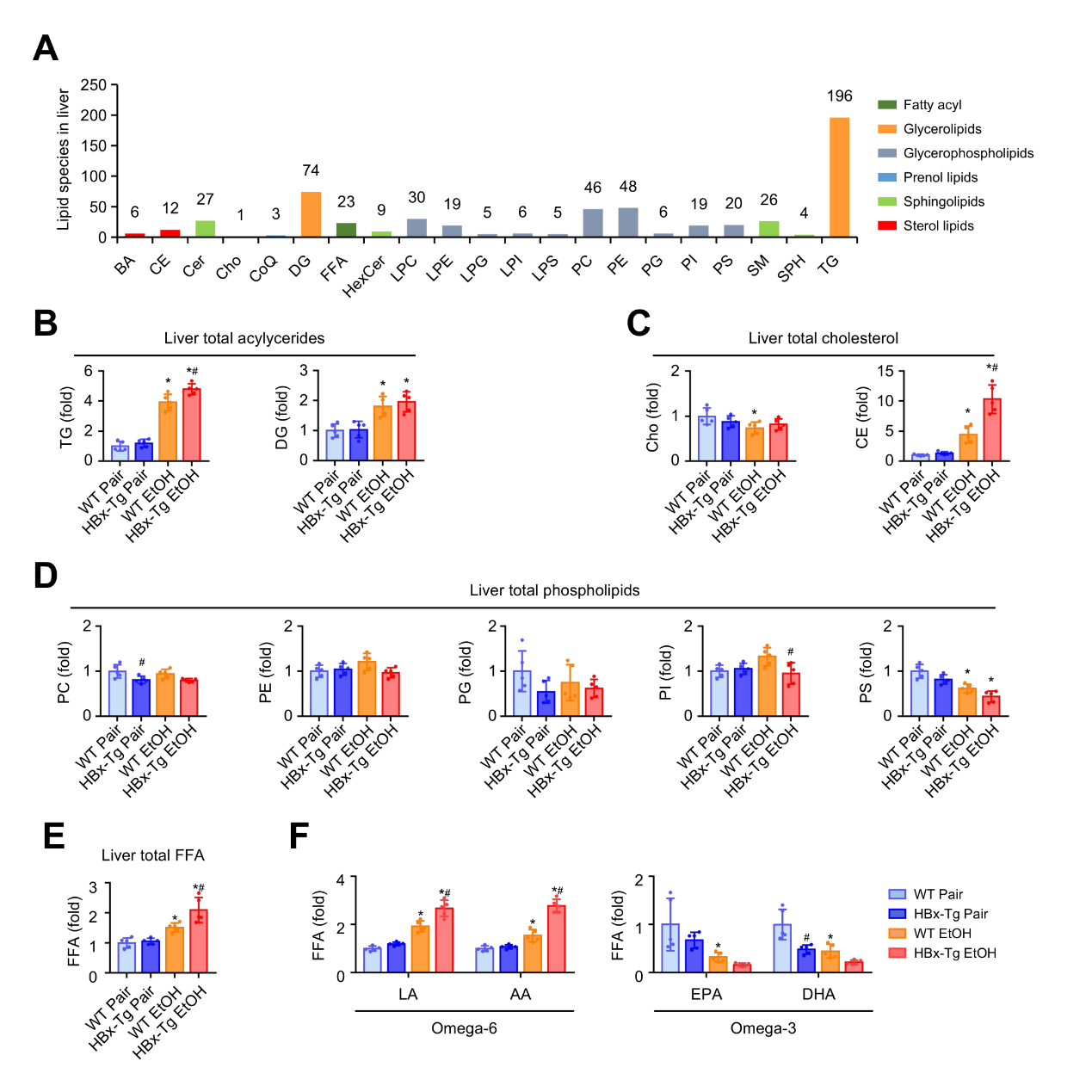


**Supplementary Figure 2. HBx induces global changes in the lipidome profile of the mouse liver.**

**(A)** Number of identified lipid species per lipid subclass. **(B)** Relative levels of total acylglycerides including TG and DG in the liver of the indicated groups. **(C)** Relative levels of total cholesterol, including Cho and CE, in the livers of the indicated groups. **(D)** Relative levels of total phospholipids, including PC, PE, PG, PI and PS, in the livers of the indicated groups. **(E)** Relative levels of total FFA in the livers of the indicated groups. **(F)** Relative levels of LA, AA, EPA and DHA in the livers of the indicated groups. Data are shown as the mean ± SD (*n* = 5 in each group). For **(B**-**F)**, ^*^*P* < 0.05 compared with the respective pair-fed group; ^#^*P* < 0.05 compared with WT ethanol-fed group; ^&^*P* < 0.05 compared with WT pair-fed group by one-way ANOVA with Tukey’s multiple comparison post-test.

LA, linoleic acid; AA, arachidonic acid; EPA, eicosapentaenoic Acid; DHA, docosahexaenoic acid.


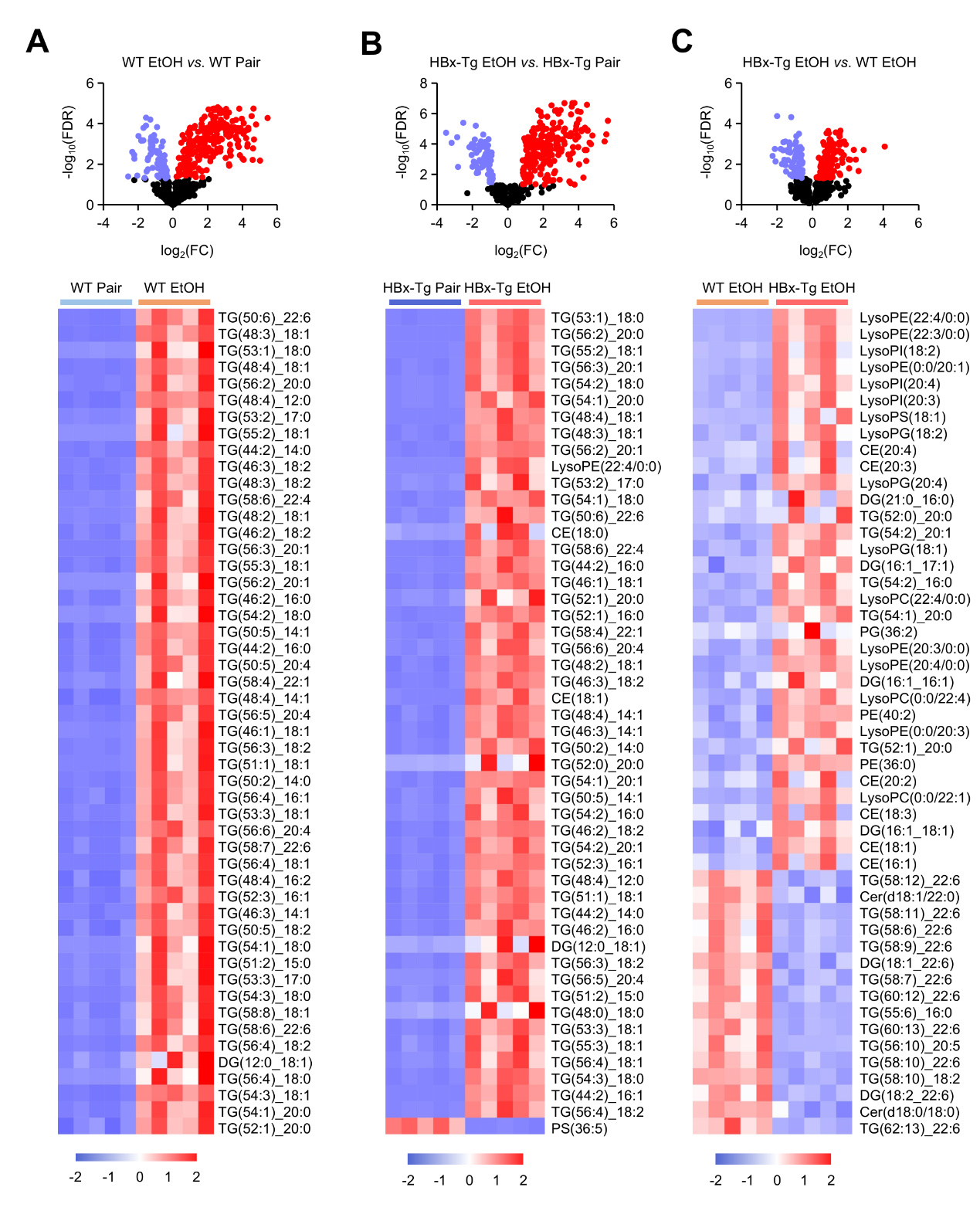


**Supplementary Figure 3. Volcano plots and heatmaps shows the differences in liver lipidomes.**

**(A)** The EtOH-fed WT mice versus the pair-fed WT mice. **(B)** The EtOH-fed HBx-Tg mice versus the pair-fed HBx-Tg mice. **(C)** The EtOH-fed HBx-Tg mice versus the EtOH-fed WT mice. For all, the volcano plots represent the log_2_ (fold change) of the indicated groups plotted against the –log_10_ (FDR). Each dot represents a specific lipid species; blue dots represent decreased lipid species; red dots represent increased lipid species and black dots represent lipid species with no significant change. Heatmaps display the top 50 differentially abundant lipid species. Rows represent individual lipid species and columns represent individual mouse samples.

FC, fold change; FDR, false discovery rate.


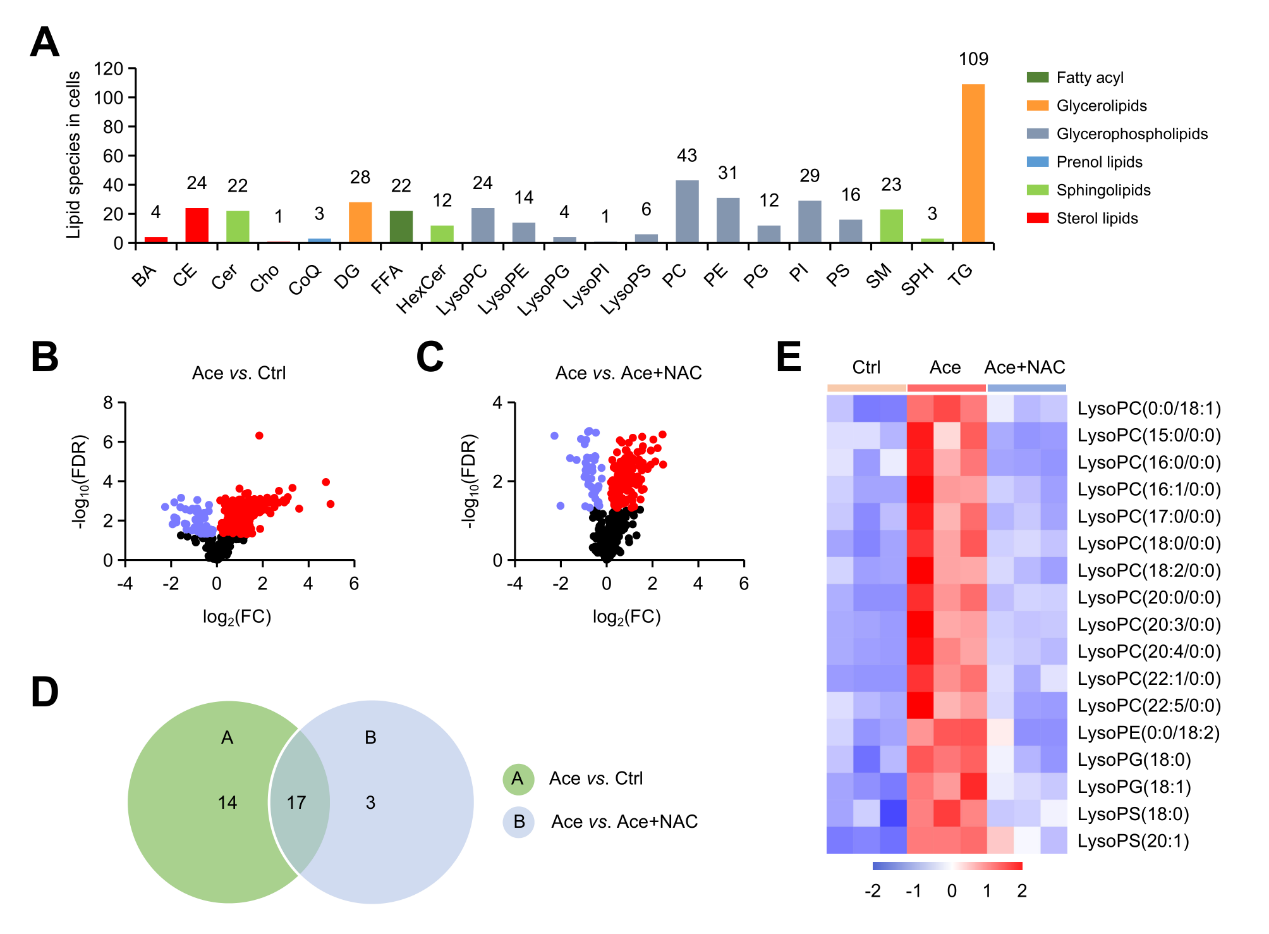


**Supplementary Figure 4. Acetaldehyde induces global changes in the lipidome profile of LO2 cells.**

**(A)** Number of identified lipid species per lipid subclass. **(B and C)** The volcano plot represents the log_2_ (fold change) of the indicated groups plotted against the –log_10_ (FDR). Each dot represents a specific lipid species; blue dots represent decreased lipid species; red dots represent increased lipid species and black dots represent lipid species with no significant change. **(D)** Venn diagram of upregulated lysophospholipids in acetaldehyde-treated cells. In total, 31 lysophospholipids were upregulated by acetaldehyde treatment. Among these lysophospholipids, 17 were rescued by NAC treatment. **(E)** Heatmap visualization of 17 selected lysophospholipids rescued by NAC treatment.


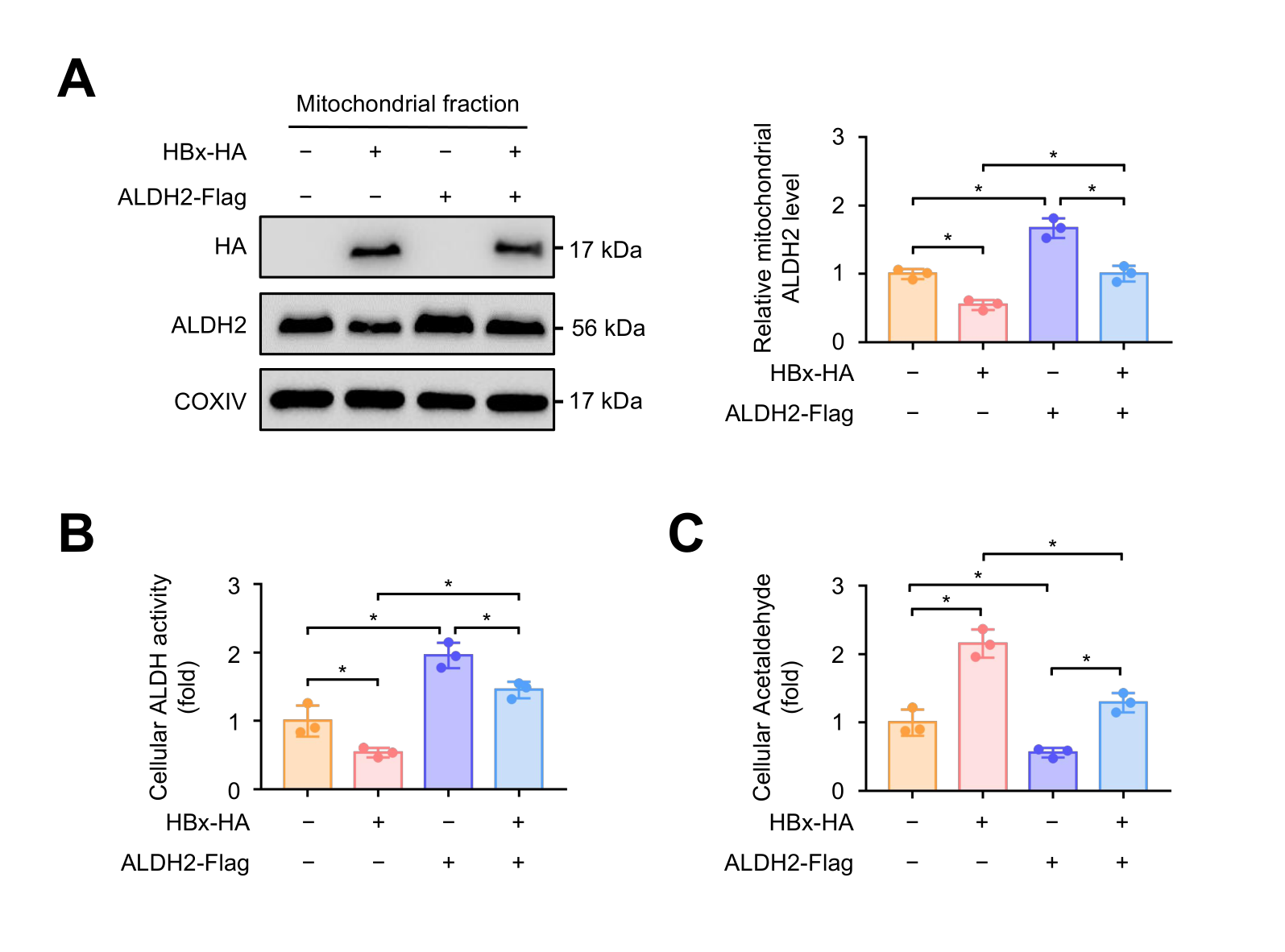


**Supplementary Figure 5. ALDH2 overexpression rescues the decreased ALDH activity in HBx-overexpressing LO2 cells.**

**(A)** LO2 cells were transfected with the indicated plasmids. Forty-eight hours after transfection, mitochondrial fractions of LO2 cells were isolated. Representative western blot images showing the indicated proteins followed by semi-quantitative analyses of the indicated groups. COXIV served as a loading control. **(B)** Relative cellular ALDH activity of LO2 cells in the indicated groups. **(C)** LO2 cells were treated with 2 mM acetaldehyde for 2 hours. Relative acetaldehyde levels of cells were analyzed. Data are shown as the mean ± SD (*n* = 3 in each group). ^*^*P* < 0.05 by one-way ANOVA with Tukey’s multiple comparison post-test.


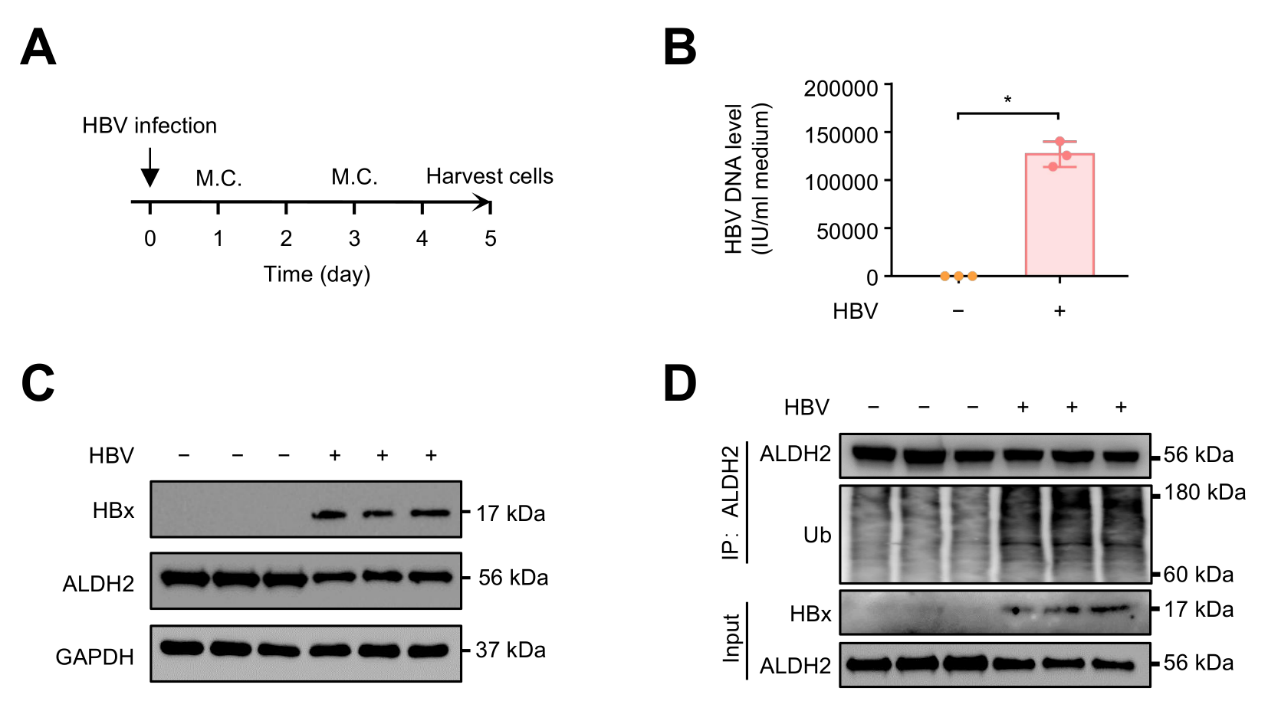


**Supplementary Figure 6. ALDH2 is downregulated in HBV-infected HepG2-NTCP cells.**

**(A)** Time course of the HBV infection experiment. M.C., medium change. **(B)** HBV DNA levels in the culture supernatants of HepG2-NTCP cells with or without HBV infection. **(C)** Western blot images showing ALDH2 and HBx protein levels in HepG2-NTCP cells with or without HBV infection. GAPDH served as a loading control. **(D)** Western blot images showing the ubiquitination of ALDH2 in HepG2-NTCP cells with or without HBV infection. Data are shown as the mean ± SD (*n* = 3 in each group), ^*^*P* < 0.05 by two-tailed Student’s t-test.
